# Targeting the p53 signaling pathway in cancers: Molecular mechanisms and clinical studies

**DOI:** 10.1002/mco2.288

**Published:** 2023-05-28

**Authors:** Jinze Shen, Qurui Wang, Yunan Mao, Wei Gao, Shiwei Duan

**Affiliations:** ^1^ Key Laboratory of Novel Targets and Drug Study for Neural Repair of Zhejiang Province School of Medicine Hangzhou City University Hangzhou Zhejiang China

**Keywords:** apoptosis, cancer biomarker, molecular mechanism, p53, targeted therapy, TRIAP1

## Abstract

Tumor suppressor p53 can transcriptionally activate downstream genes in response to stress, and then regulate the cell cycle, DNA repair, metabolism, angiogenesis, apoptosis, and other biological responses. p53 has seven functional domains and 12 splice isoforms, and different domains and subtypes play different roles. The activation and inactivation of p53 are finely regulated and are associated with phosphorylation/acetylation modification and ubiquitination modification, respectively. Abnormal activation of p53 is closely related to the occurrence and development of cancer. While targeted therapy of the p53 signaling pathway is still in its early stages and only a few drugs or treatments have entered clinical trials, the development of new drugs and ongoing clinical trials are expected to lead to the widespread use of p53 signaling‐targeted therapy in cancer treatment in the future. TRIAP1 is a novel p53 downstream inhibitor of apoptosis. TRIAP1 is the homolog of yeast mitochondrial intermembrane protein MDM35, which can play a tumor‐promoting role by blocking the mitochondria‐dependent apoptosis pathway. This work provides a systematic overview of recent basic research and clinical progress in the p53 signaling pathway and proposes that TRIAP1 is an important therapeutic target downstream of p53 signaling.

## Graphical abstract

Activated p53 signaling has important roles, including antiangiogenic, cell cycle arrest, promotion of DNA repair, regulation of cellular metabolism, and promotion of apoptosis. A novel p53‐dependent downstream gene, TRIAP1, inhibits the mitochondria‐dependent apoptotic pathway. This work systematically describes the molecular regulatory mechanism of p53 signaling and suggests that TRIAP1 has potential therapeutic value.

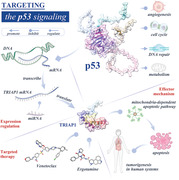

## 1 INTRODUCTION

The tumor‐suppressor protein p53 is known as the guardian of the genome. p53 is involved in the activation of various biological responses, mainly including cell cycle arrest, DNA repair, and apoptosis.^1^, ^2^, ^3^, ^4^ Activation of p53 is mediated by multiple stress signals, including hypoxia, DNA damage, and strong proliferative signals.^5^, ^6^, ^7^, ^8^ Inhibition of MDM2 and its cognate complex protein MDMX is the most important regulatory pathway for p53 activation.^9^ Stress signals can upregulate the expression of ATM, ATR, and other proteins through sensory proteins, and reduce the expression of MDM2.^10^, ^11^ When the proto‐oncogene is activated, ARF is activated and the inhibition of MDM2 expression is enhanced.^12^, ^13^ Second, some upstream factors can also directly activate p53, for example, ATM and ATR proteins can promote the phosphorylation of p53 through CHK1/CHK2 or directly and increase the expression level of p53.^14^, ^15^ Dysregulation of p53 function can be detected in approximately 90% of cancers, including TP53 mutations or abnormal activation of other upstream factors.^16^, ^17^ Stress signals such as DNA damage, cellular hypoxia, reactive oxygen species (ROS) damage, and activation of proto‐oncogenes increase the amount and activity of p53 after transcription by promoting p53 phosphorylation and inhibiting p53 ubiquitination.^18^, ^19^, ^20^, ^21^, ^22^ Activated p53 forms a tetramer and binds to a variety of target genes and promotes their transcription, thereby inhibiting cell carcinogenesis.^7^, ^23^, ^24^

Abnormalities in the p53 pathway interfere with the normal cell cycle, programmed cell death, DNA repair, senescence, and angiogenesis, ultimately leading to cancer progression.^25^, ^26^, ^27^, ^28^, ^29^, ^30^ Restoring normal expression and activity of p53 is a potential strategy for the treatment of various cancers.^31^ Currently, many compounds that can reactivate p53 or destabilize mutant p53, such as APR‐246 and COTI‐2, have been approved for clinical trials.^32^ At the same time, MDM2 inhibitors have also been related to research, and inhibition of MDM2–p53 interaction has become a possible therapy.^33^, ^34^, ^35^, ^36^, ^37^ However, the corresponding research has mainly focused on the mutation site of TP53 or p53, and the relevant clinical transformation is still in its infancy.^38^ Although different mutant p53 (mut‐p53)‐targeted therapeutic strategies have been developed, there are still no approved drugs for the clinical treatment of mut‐p53‐expressing cancers.^39^ At the same time, as a nuclear transcription factor, p53 does not have typical drug target characteristics, so it has long been considered untreatable. Although many p53‐based therapies have been explored and studied in the past three decades, few p53 drug development programs have reached the advanced clinical trial stage, and none of them have been approved by the United States Food and Drug Administration until now.^40^

TP53‐regulated inhibitor of apoptosis 1 (TRIAP1) is a novel p53 downstream gene that mediates the antiapoptotic activity of p53. TRIAP1 expression is tightly regulated by p53, and phosphorylation at different sites on p53 can significantly affect TRIAP1 expression.^41^ The translation process of TRIAP1 is regulated by various noncoding RNAs (ncRNAs).^42^ Numerous studies have shown that TRIAP1 is a novel tumor diagnostic and prognostic marker. TRIAP1 is abnormally highly expressed in a variety of tumors and has a cancer‐promoting effect.^43^ TRIAP1 expression was significantly higher in multiple drug‐resistant cancer cell lines (doxorubicin (DOX), cisplatin (DDP), tamoxifen (TAM), and etoposide (VP‐16)) than in corresponding drug‐sensitive cell lines.^44^ In addition, as an independent prognostic factor, TRIAP1 overexpression often predicts poorer overall survival (OS) and disease‐free survival (DFS) in patients, and more advanced histological grade.^45^

Despite the difficulties, targeting the p53 pathway remains a top priority in cancer research. Therefore, we systematically classified the important or new gene targets in the p53 pathway in recent years and reviewed their molecular mechanisms in the p53 pathway. At the same time, our work also sorts out the clinical value of these gene targets and the corresponding targeted therapy research. Finally, this study outlines the shortcomings of current related research, aiming to provide direction and a basis for further in‐depth research.

## 2 THE STRUCTURE, MUTATION, AND MODIFICATION OF p53

**FIGURE 1:**
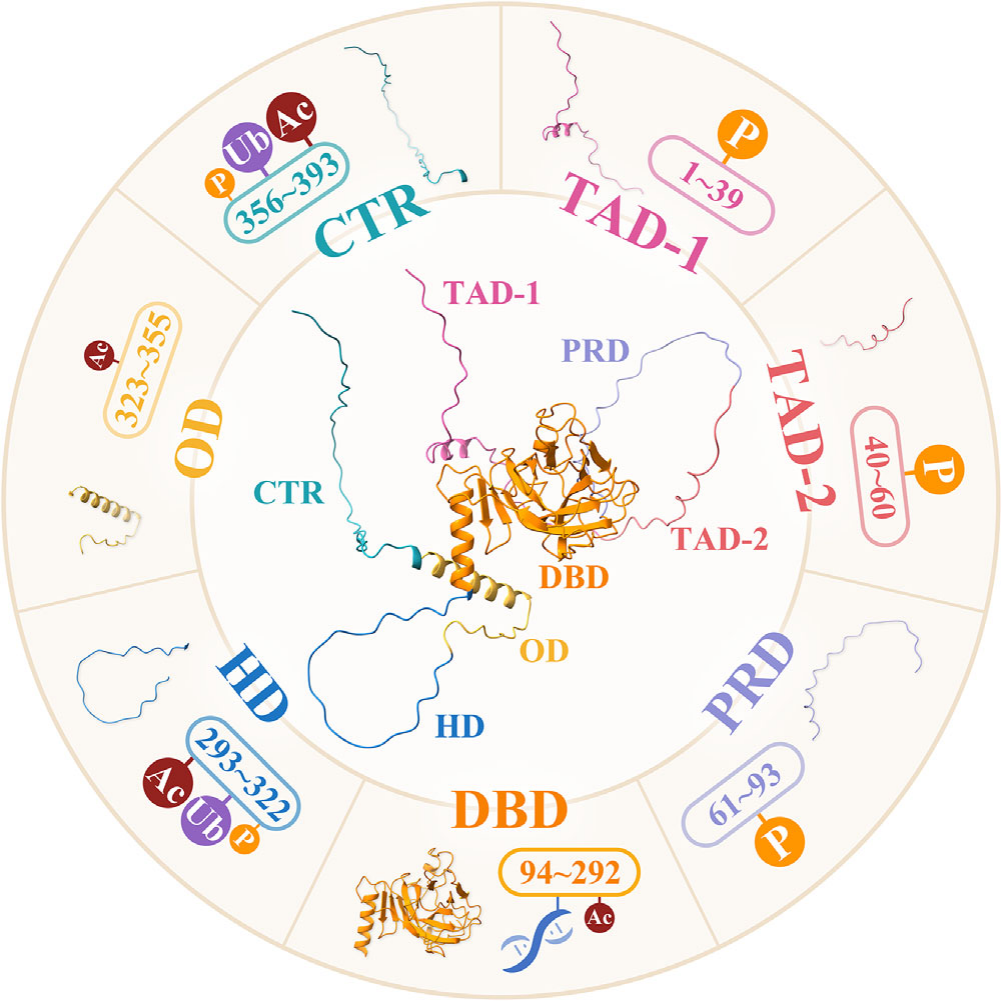
Functional domains of p53. p53 consists of 393 amino acids, which are usually divided into seven functional domains from N‐terminal to C‐terminal, including TAD‐1 (amino acid 1–39), TAD‐2 (amino acid 40–60), PRD (amino acid 61–93), DBD (amino acid 94–292), HD (amino acid 293–322), OD (amino acid 323–355), and CTR (amino acid 356–393). Phosphorylation (P) occurs in TAD‐1, TAD‐2, and PRD, and acetylation (Ac) and ubiquitination (Ub) occur in HD and CTR. DBD can bind DNA and is the domain of p53 to exert transcriptional activity.

p53 is a specific transcription factor consisting of 393 amino acids with 7 functional domains.^46^, ^47^ p53 is encoded by the TP53 gene, which includes 11 exons and 10 introns. As shown in Figure 1, p53 is divided into 7 functional domains from N‐terminal to C‐terminal, transactivation domain (TAD) ‐1, TAD‐2, proline‐rich domain (PRD), DNA‐binding domain (DBD), hinge domain (HD), oligomerization domain (OD), and C‐terminal regulatory domain (CTR).^47^, ^48^

For a long time, p53 was considered to be the only expression product of the TP53 gene. However, under physiological conditions, 12 p53 subtypes have been discovered so far, and each subtype uses different promoters, splicing events, and alternative translation initiation sites to distinguish each other.^49^, ^50^ The p53 isoforms are divided into α, β, and γ according to the difference of the C‐terminus. Among them, the α class is the longest, and it contains all the domains of the C‐terminus of p53.^51^ The β class retains the α nuclear localization sequence, and at the same time adds a unique sequence of 10 amino acids derived from exon 9b, but deletes most of the OD domain after amino acid 331 and the entire CTR domain.^52^ The γ class also lacks the sequence after amino acid 331 and correspondingly increases the unique sequence of 15 amino acids derived from exon 9b.^53^

According to the amino acid sequence of the N‐terminal deletion, the p53 subtypes are divided into four types: TAp53, Δ40p53, Δ133p53, and Δ160p53. TAp53 is the complete p53, while Δ40p53 is the result of alternative splicing of the original p53, which lacks the first 39 amino acids and therefore lacks the TAD‐1 domain, but still retains all the TAD‐2 and DBD domains.^54^ The Δ133p53 isoform lacks the first 132 amino acids and thus lacks TAD‐1, TAD‐2, PRD, and part of the DBD.^55^ The Δ160p53 isoform lacks the first 159 amino acids and thus lacks TAD‐1, TAD‐2, PRD, and more DBD regions.^56^ Deletions and changes between subtypes will affect the function of the p53 protein, for example, the subtypes missing the C‐terminus will affect the orientation of p53 to target genes. p53β enhances the transactivation of p21 and Bax promoters in breast cancer (BrC). In contrast, p53γ can only stimulate the transactivation activity of the Bax promoter.^57^ Isoforms lacking the N‐terminus tend to have a reduced ability to activate transcription of p53 target genes due to loss of TAD. However, Δ133p53 and Δ160p53 further delete the ubiquitination site at the N‐terminus, which reduces the proteasome degradation rate and improves stability.^49^, ^58^ At the same time, complexes can also be formed between different p53 subtypes to positively or negatively regulate p53‐dependent gene expression.^1^

### 2.1 Functional changes and prognostic value of mutant p53

About 50% of cancers with confirmed p53 inactivation are caused by point mutations in the TP53 gene.^16^, ^39^ Most of them are missense mutations in DBD, which make p53 lose its DNA binding activity, thereby promoting tumor development.^59^ For example, in diffuse large B‐cell lymphoma (DLBCL), p53 mutations are associated with poorer OS and PFS.^60^ Similarly, missense mutations in TAD often lead to poor prognosis in cancer patients. For example, in H1299 cells, mut‐TAD (L22Q and W23S) can inhibit the phosphorylation of p53 Ser20, weaken the activation of p53 by PLK3, and make p53 occur Dominant oncogenic alteration.^61^ There are also some mutations and some mutations only make p53 easy to aggregate, eventually forming inactive amyloid aggregates. At this time, if the aggregation of p53 proteins can be prevented, part of the function of p53 can be preserved.^62^

Missense mutations in the p53 DBD also affect the interaction of p53 with other proteins. For example, mut‐DBD (R248Q and R249S) can lead to an enhanced antifolding effect of Hsp70/Hdj1 on mut‐p53, making p53 more susceptible to breakdown by Hsp70/Hdj1. The DNA‐binding loop dissociates, leaving p53 in a hypoactive, deprotected state. However, mut‐DBD‐R175H attenuates Hsp70/Hdj1 antifolding and stabilizes p53 from degradation.^63^

But now more and more studies have found that some nondestructive mutations in p53 DBD can be used as favorable factors for cancer prognosis. For example, partial missense mutations in the p53 DBD have been associated with prolonged PFS in metastatic colorectal cancer (mCRC) patients with bevacizumab‐based therapy.^64^ Missense mutations in DBD (including R273X, Y220X, H193, H179X, and R175H) are associated with prolonged OS and PFS in resectable esophageal squamous cell carcinoma (ESCC).^65^ Other mutations in the DBD have vastly different effects on p53 function. It is speculated that this may be due to nondestructive mutations that lead to structural changes in DBD and enhanced transcriptional activity of some tumor suppressor genes, leading to enhanced sensitivity of cancer cells to treatment.^64^ However, the mechanism still needs more prospective research to discover and confirm.

### 2.2 Posttranslational modifications of p53

An additional ∼40% of cancers with confirmed p53 inactivation were due to alterations in p53 regulators.^66^ This process involves a variety of posttranslational modifications, such as phosphorylation, acetylation, ubiquitination, and other modifications, to jointly regulate the activity and stability of p53.^67^, ^68^, ^69^

#### 2.2.1 Phosphorylation modifications of p53

Phosphorylation mostly occurs at Ser6, Ser9, Ser15, Thr18, Ser20, Ser33, Ser37, Ser46, Thr55, and Thr81 on the p53 N‐terminal domain TAD‐1 and TAD‐2.^70^, ^71^ Other sites on p53 can also be phosphorylated, such as Ser313, Ser314, Ser315, Ser366, Ser376, Thr377, Ser378, Thr387, and Ser392.^72^, ^73^ The phosphorylation of p53 can be mediated by various protein kinases, including ataxia telangiectasia mutated (ATM), ataxia telangiectasia and Rad3‐related protein (ATR), DNA‐dependent protein kinase (DNA‐PK), checkpoint kinase‐1 (Chk1), checkpoint kinase‐2 (Chk2), and c‐Jun N‐terminal kinase.^13^, ^74^, ^75^, ^76^, ^77^ The protein phosphatase magnesium‐dependent 1 delta mediates the dephosphorylation process of p53.^78^

Phosphorylation increases the stability of p53 through multiple mechanisms. For example, phosphorylation on Thr81 changes the conformation of p53 and inhibits the binding of p53 to the ubiquitinase MDM2.^79^ Phosphorylation can also improve the affinity of p53 to the acetylase p300/CRB and compete for the lysine site on p53 through acetylation and ubiquitination, thereby improving the stability of p53 and facilitating the biological function of p53.^18^, ^80^ At the same time, depending on the position of the phosphorylated residue, the orientation of p53 binding to target genes will be changed. For example, the phosphorylation of p53 Ser46 is closely related to the induction of apoptosis.^81^ Phosphorylation of p53 Ser42 plays an important role in cell survival after DNA damage.^82^

Many studies have confirmed that the phosphorylation site of p53 can affect the prognosis and pathological conditions of cancer patients. In general, patients with high levels of p53 phosphorylation have a better prognosis. Hepatocellular carcinoma (HCC) patients with high levels of p53 Ser46 phosphorylation have relatively longer OS.^83^ BCa patients with high p53 Thr81 phosphorylation have relatively longer OS with lower grade and lower tumor size.^84^ BrC, CRC, and glioma patients with high levels of p53 Ser366 phosphorylation have relatively longer OS.^72^ HCC patients with high levels of p53 Ser392 phosphorylation have relatively longer OS and recurrence‐free survival (RFS).^85^ In contrast, BCa and non‐small‐cell lung cancer (NSCLC) patients with high levels of p53 Ser315 phosphorylation have relatively shorter OS and later TNM stage and histological grade.^86^, ^87^

#### 2.2.2 Acetylation modifications of p53

Acetylation of p53 occurs on the lysine residues in the p53 CTR domain, including Lys101, Lys120, Lys139, Lys164, Lys305, Lys319, Lys320, Lys370, Lys372, Lys373, Lys381, Lys382, Lys386, and so on. This is followed by lysine residues in the OD and DBD domains.^88^, ^89^ p53 acetylation can be promoted by acetyltransferases such as p300/CREB‐binding protein (CBP), p300/CBP‐associated factor (PCAF), and 60 kDa taf interactive protein (TIP60),^18^, ^90^, ^91^ but can also be blocked by deacetylases such as histone deacetylase (HDAC) and Sirtuin (SIRT).^92^, ^93^

The acetylation of p53 can be activated by factors such as DNA damage and genotoxic stress, which is similar to phosphorylation. Phosphorylation of p53 can increase the affinity of p53 to the acetylase p300 and then significantly promote acetylation.^94^ The acetylation of p53 can compete with ubiquitination, and the acetyl group competes with ubiquitin for lysine residues on the CTR, which can improve the stability of p53 and greatly activate the activity of the DBD domain.^88^, ^95^ The site and degree of p53 acetylation will affect the function of p53. For example, acetylation of both Lys120 and Lys164 on the DBD domain of p53 helps induce cell cycle arrest, whereas acetylation of Lys120 alone tends to induce apoptosis.^96^

Many studies have now confirmed that multiple p53 acetylation sites can also affect the prognosis and pathological status of cancer patients. Likewise, patients with high p53 and high acetylation levels had a better prognosis. For example, HCC patients with high levels of p53 Lys120 acetylation have relatively longer OS.^97^ Malignant melanoma and HCC patients with high levels of p53 Lys373 acetylation have relatively longer OS and early, lower lymph node metastasis.^98^, ^99^ HCC, malignant melanoma, and lung cancer patients with high levels of p53 Lys382 acetylation have relatively longer OS and earlier stage and lower lymph node metastasis.^98^, ^99^, ^100^

#### 2.2.3 Ubiquitination and ubiquitin‐like modifications of p53

p53 ubiquitination is the same as acetylation, and it also mainly occurs on lysine residues of p53, including Lys305, Lys319, Lys320, Lys370, Lys372, Lys373, Lys381, Lys382, and Lys386. p53 ubiquitination is mainly mediated by ubiquitinases such as mouse double minute 2 (MDM2), mouse double minute 4 (MDM4), COP1, Pirh2, and Arf‐BP1.^101^, ^102^, ^103^, ^104^ However, deubiquitinating enzymes such as USP11 and UCH‐L1 can mediate p53 deubiquitination.^105^, ^106^ Different levels of ubiquitination have different subsequent effects on p53, for example, high levels of ubiquitination directly mediate the degradation of p53 by the proteasome. When the ubiquitination level is low, it can inhibit the transcriptional activity of p53 by competing with acetyl to bind to the lysine residue of the C‐terminal domain or transfer p53 to the mitochondria, preventing the binding between p53 and target genes in the nucleus.^88^, ^107^

Ubiquitin‐like proteins are a family of proteins with a structure similar to ubiquitin and a close evolutionary relationship, mainly including small ubiquitin‐like modifier (SUMO), neural precursor cell expressed developmentally downregulated protein 8 (NEDD8), ATG8, ATG12, URM1, UFM1, FAT10, and ISG15.^108^ Among them, SUMO and NEDD8 are closely related to p53 modification.^109^ The SUMO is a small ubiquitin‐like molecule.^110^ The sumoylation of p53 is mainly mediated by specific SUMO‐E3‐ligase such as TOP1 binding arginine/serine‐rich protein (Topor) and protein inhibitor of activated stat.^111^, ^112^ Compared with ubiquitination, sumoylation of p53 does not promote the degradation of p53 but inhibits the transcriptional activation of p53 on target genes by competing with acetyl for lysine residues in CTR.^113^ NEDD8 is a ubiquitin‐like small molecule with a high similarity to ubiquitin.^114^ NEDDylation can not only directly inhibit the transcriptional activity of p53, but also prevent the degradation of MDM2, and indirectly promote the ubiquitination of p53 by MDM2.^18^

#### 2.2.4 Epigenetic modifications of p53

Epigenetics refers to changes in heritable gene expression or cell phenotype caused by mechanisms that do not alter the DNA sequence.^115^ Many macromolecules in organisms undergo chemical modifications that serve crucial functions.^116^ Epigenetic modification, which alters gene expression by introducing chemical modifications, is an important form of epigenetic regulation. This includes histone modification, DNA modification, and RNA modification.^117^ TP53‐associated H3K9 and H3K14 acetylation promotes TP53 transcription,^118^ while monomethylation on H4K20 represses TP53 transcription by inhibiting nucleosome depolymerization.^119^, ^120^ High levels of H2B ubiquitination are often associated with DNA repair.^121^, ^122^ High methylation levels in the TP53 promoter region can hinder the binding of transcription factors and inhibit TP53 expression, leading to various cancers.^123^ DNA methyltransferase inhibitors such as 5‐aza‐2'‐deoxycytidine can downregulate methylation levels in the TP53 promoter region, facilitating the binding of transcription factors and promoting TP53 expression.^124^ Modifications in mRNA can affect its stability, readability, and translation efficiency,^125^ and m6A modification in TP53 mRNA can upregulate p53 expression.^126^ Nm modification on rRNA^127^ and ψ modification on snRNA^128^ can indirectly affect p53 expression. Epigenetic regulation complexly and precisely regulates the transcription and translation of the TP53 gene, affecting p53 levels and participating in various disease processes, particularly cancer regulation.^129^ However, research on p53‐related epigenetics is still limited and there are many gaps in our understanding of how epigenetic modification affects p53 expression. These include novel histone modifications such as histone sulfation,^130^ histone lactylation,^131^ and histone lysine benzoylation^132^; novel DNA methylation modifications such as 3‐methylcytosine (3mC),^133^ N4‐methylcytosine (4mC),^134^ N6‐methyladenine (6mA),^135^ and 7‐methylguanosine (7mG)^136^; and mRNA modifications such as N1‐methyladenosine (m1A) and N4‐acetylcytidine (ac4C). With the rapid advancement of epigenetic sequencing technology, we can expect many breakthrough discoveries in the field of p53‐related epigenetic modification in the near future.

## 3 ACTIVATION AND INACTIVATION OF p53

**FIGURE 2:**
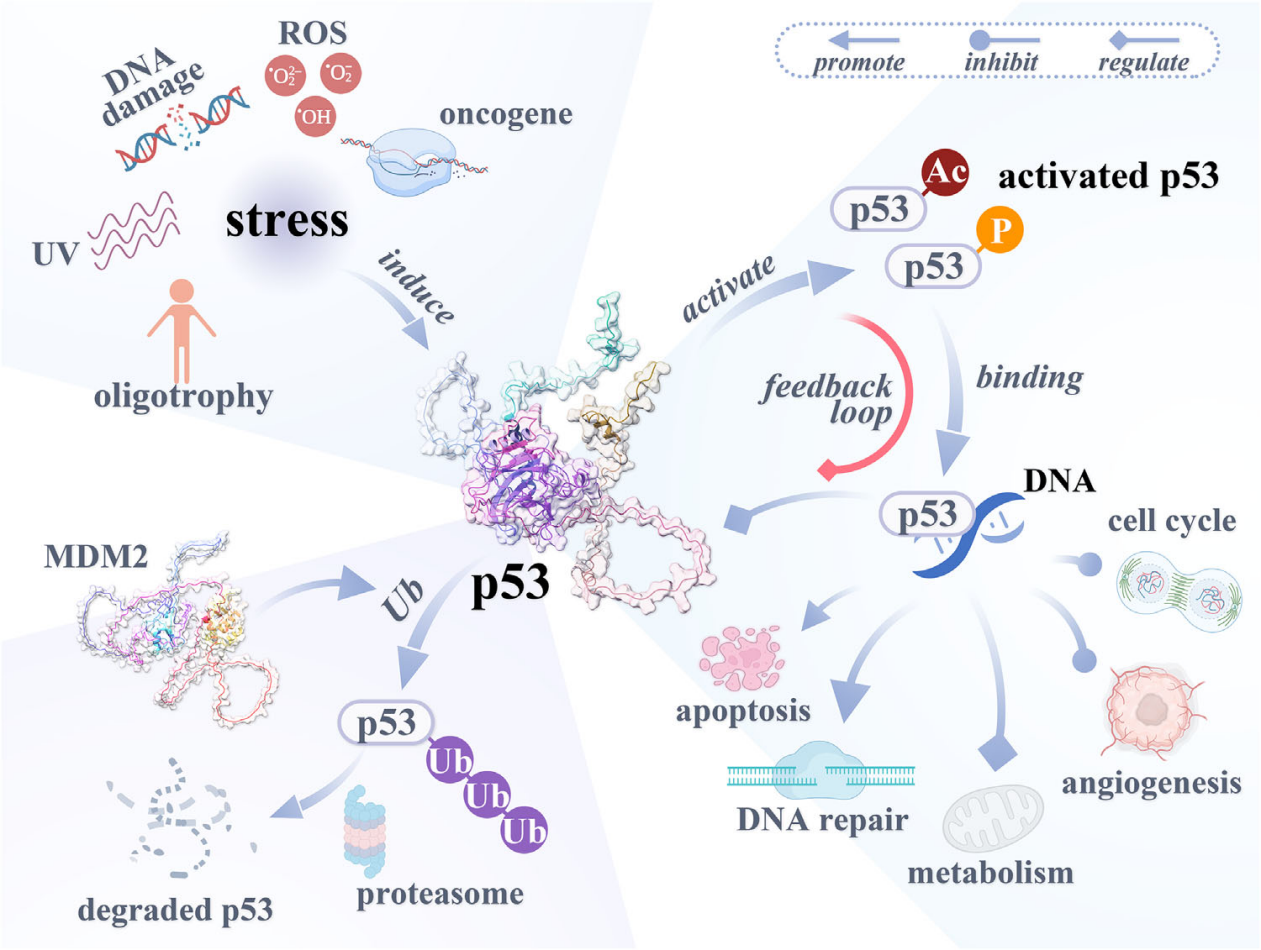
The function of p53 and its regulation mechanism. p53 can be activated under various stresses, including oligotrophy, UV radiation, DNA damage, ROS, activation of an oncogene, and so on. Activated p53 can bind and induce target gene transcription on DNA, thereby promoting apoptosis and DNA repair, inhibiting cell cycle and angiogenesis, and regulating various metabolisms. Some downstream target genes of p53 are also involved in the regulation of p53 levels, thereby forming a feedback loop. MDM2 is a partner protein of p53, which ubiquitinates p53 and subsequently causes p53 to be degraded by the proteasome. UV, ultraviolet; ROS, reactive oxygen species; Ac, acetylation; P, phosphorylation; Ub, ubiquitination.

The activation and inactivation of p53 are precisely regulated by many factors. As shown in Figure 2, the activation of p53 mainly involves biological mechanisms such as acetylation and phosphorylation, while the inactivation of p53 is closely related to its ubiquitination, ubiquitination, and partial dephosphorylation.^1^, ^137^ At the same time, various proteins are not only activated by the transcription of p53 but also participate in the regulation of p53 activation or inactivation.^138^ p53 and these proteins constitute a complex feedback regulatory network, including Siva1,^139^ COP1,^140^ Pirh2,^140^ cyclin G,^141^ ΔNp73,^142^ and so on, as well as the classic MDM2.^143^

### 3.1 p53 activation‐related factors

ATM protein and ATR both belong to the ataxia telangiectasia (A‐T) family of proteins, but their timing of effects differs. ATM is activated by acetylation of the 60 kDa tat‐interacting protein (TIP60) at DNA double‐strand breaks (DSBs). ATM activates p53, checkpoint kinase 2 (CHK2), and other responsive proteins through phosphorylation, mediating subsequent cell cycle arrest, DNA repair, apoptosis, and other biological processes.^144^ ATR is activated when various DNA single‐strand damage occurs, and it plays a similar role to ATM through phosphorylation and activation of p53, checkpoint kinase‐1 (CHK1), and other reactive proteins.^145^ CHK1 and CHK2 are cyclin‐dependent kinase (CDK) proteins, which are phosphorylated and activated by ATR and ATM, respectively, thereby acting as phosphokinases to activate p53 and CDC25A and other proteins.^146^, ^147^

DNA‐PK is a nuclear serine/threonine protein kinase consisting of a catalytic subunit (DNA‐PKcs) and a DNA targeting subunit (Ku).^148^ Ku has a high affinity to the end of defective DSB and can recruit DNA‐PKcs to the DSB region, triggering the activation of phosphoinositide 3‐kinase‐related kinase family protein DNA‐PKcs. Subsequently, it phosphorylates many substrates, including DNA‐PKcs itself, XRCC4, and Artemis, and other proteins, including p53, thereby playing a regulatory role in transcription, replication, and DNA repair.^149^, ^150^

p300/PCB and p300/PCAF are GNAT super‐family proteins, which have the function of acetylating p53, protecting p53 from ubiquitination, thereby promoting the transcriptional activation of p53.^151^, ^152^ Both TIP60 and MOF are members of the MYST family and were found earlier to have the function of acetylating histone lysine residues.^153^ Recent studies have found that TIP60 and MOF have the same ability to acetylate lysine residues on p53, which can promote the transcriptional activation ability of p53 and avoid its ubiquitination.^154^, ^155^

Multiple p53 negative regulators, including MDM2 and ArfBP1, are inactivated by alternate reading frame (ARF) targeting, which is common during nucleolar stress.^156^ The nucleolus is a large organelle without a nuclear membrane and is primarily responsible for the biogenesis of ribosomes. The shape, size, and number of nucleoli are directly related to ribosome biogenesis.^157^ When errors in steps of ribosomal biogenesis are sensed by the nucleolus, they trigger nucleolar stress, leading to global changes in nucleolar function and morphology. Nucleolar stress can lead to the inactivation of negative regulatory molecules such as p53, promote p53 stabilization, and then activate cell cycle arrest or apoptosis.^158^

The tumor suppressor protein phosphatase and tensin homolog (PTEN) is transcriptionally activated by p53 and has dual‐specificity phosphatase activity, namely protein phosphatase activity and lipid phosphatase activity.^159^ PTEN can inhibit the phosphorylation and nuclear translocation of MDM2 by inhibiting the PI3K/AKT signaling pathway.^160^, ^161^ Activated p53 can induce PTEN to limit the regulation of MDM2 ubiquitination of p53 in the nucleus, thereby greatly increasing the level of p53 in the nucleus. This positive feedback regulation is beneficial to the repair of damage or induces apoptosis of irreversibly damaged cells.^162^

### 3.2 p53 inactivation‐related factors

MDM2 exists as a partner of p53 and belongs to the E3 ubiquitin‐ligase class. The C‐terminal zinc finger domain of MDM2 has the ability to ubiquitinate p53 and induce proteasomal degradation of ubiquitinated p53. The N‐terminal domain of MDM2 can also bind to the transcriptional activation domain of p53 by forming a hydrophobic domain and inhibiting its function.^18^, ^163^ p53 can also target the MDM2 gene and promote MDM2 protein expression. Under normal physiological conditions, a closed‐loop negative feedback regulation is formed between MDM2 and p53, and p53 is maintained at a low level.^18^ However, during acute stress, p53 can induce MDM2 to inhibit the expression of various downstream proapoptotic proteins through ubiquitination, providing repair opportunities for damaged cells.

The level of MDM2 is regulated by a variety of proteins, which indirectly affect the activity of p53, including MDM2 itself, SCYL1‐BP1,^164^ p14ARF,^165^ TRIM8,^166^ and so on. In addition, self‐ubiquitination of MDM2 reduces MDM2 levels, which in turn keeps p53 stable.^167^ SCYL1‐BP1 can bind to inhibit MDM2 and promote MDM2 autoubiquitination and has a strong inhibitory effect on MDM2.^168^ The tumor suppressor gene p14ARF is activated when the proto‐oncogenes C‐MYC, Ras, and E2F1 are activated, and has the ability to inhibit the metastasis of MDM2, thereby inhibiting the ubiquitination of p53 by MDM2.^8^, ^169^, ^170^, ^171^ TRIM8 has the ability to bind and inhibit MDM2, can replace p53 and bind to MDM2, and can also degrade MDM2, thereby inhibiting the ubiquitination of p53 by MDM2.^166^ MDM2 is also modified by small ubiquitin‐like molecules. For example, NEDD8 can compete with ubiquitin to bind to MDM2 protein, inhibit the self‐ubiquitination process of MDM2, stabilize MDM2, and promote the degradation of p53.^172^ SUMO has a zinc finger domain that binds MDM2 and inhibits the ubiquitination of p53 by MDM2.^173^ Exogenous factor nutlin‐3 is an exogenous nongenotoxic activator of the p53 pathway, which can indirectly activate the p53 pathway by antagonizing MDM2. The p53 pathway products induced by nutlin‐3 are mainly p21 and PUMA, and play a role in cell cycle arrest and antiangiogenesis.^174^, ^175^, ^176^

MDMX and MDM2 belong to the E3 ubiquitin‐ligase class and have a similar structure to MDM2.^177^ MDMX only has the ability to bind and inhibit p53 but lacks the ability to induce the degradation of p53. However, MDMX can bind to MDM2 as a heterodimer to stabilize MDM2, which promotes the ubiquitination of p53 by MDM2.^178^, ^179^

p53 can target and upregulate COP1, Pirh2, and ArfBP1, and this promotes their ubiquitination and degradation of p53, thereby forming a similar negative feedback regulatory system.^180^, ^181^, ^182^ COP1 also has a mechanism similar to MDM2's self‐ubiquitination. The self‐ubiquitination of COP1 can be activated by ATM phosphorylation, thereby weakening the inhibitory effect on p53 when cells are stressed by external stress.^180^, ^183^

SIRT1 is a nicotinamide adenine dinucleotide‐dependent sirtuin located upstream of p53, which inhibits the transcriptional activation ability of p53 through the deacetylation of p53.^184^ The high expression of SIRT1 plays an important role in the metastasis of various cancers, including prostate cancer (PCa) and ESCC.^185^

Leucine zipper‐containing ARF‐binding protein (LZAP) is down‐regulated in various cancer cells. Downregulation of LZAP expression is associated with enhanced invasion, xenograft tumor growth, and angiogenesis.^186^ LZAP is generally considered a tumor suppressor, which can inhibit the high level of p53, and mainly plays a tumor suppressor role after p53 mutation or deletion.^187^ LZAP is also positively correlated with the function of wild‐type p53‐induced phosphatase 1 (WIP1), which can promote the binding of WIP1 to various substrates.^188^ WIP1 belongs to the protein phosphatase 2C (PP2C) family, a mammalian serine/threonine‐specific protein phosphatase.^189^ WIP1 can be targeted and activated by p53, and can also dephosphorylate a variety of DNA damage response pathway signaling molecules including p53, which is closely related to the occurrence of cancer.^190^, ^191^ In general, the phosphorylation at the Ser15 site of p53 can protect itself from being ubiquitinated by binding to MDM2, and WIP1 can remove the phosphorylation at the Ser15 site of p53, thereby restoring the ubiquitination of p53 by MDM2.^192^ A recent study shows that the complex formed by p53 and p21 can increase the sensitivity of p53 to WIP1 and MDM2, thereby promoting the dephosphorylation and ubiquitination of p53 to form a negative feedback regulatory loop.^137^

## 4 REGULATORY MECHANISMS OF p53 On Downstream Genes

As shown in Figure 2, activated p53 can transcriptionally activate a variety of downstream effector proteins, and play an important role in cell cycle inhibition, DNA damage repair, apoptosis promotion, inhibition of angiogenesis and metastasis, and immune regulation.^40^ The functions of the target genes activated by p53 are complex and diverse, and most of them have the function of suppressing tumors, but there are also a small number of target genes that can promote the development of cancer.^193^, ^194^

### 4.1 Regulation of p53 on cell cycle

Cell cycle checkpoints are important molecular mechanisms in the cell cycle to ensure the quality of DNA replication and chromosome allocation.^195^ When abnormal events occur during the cell cycles process, such as DNA damage or DNA replication blockage, the cell cycle checkpoint is activated to interrupt the operation of the cell cycle in time. The cell cycle cannot resume until the cell is repaired.^195^ Uncontrolled cell division or dissemination of damaged DNA can lead to genomic instability and tumorigenesis.^196^ p53 can induce factors such as p21, GADD45A, and 14‐3‐3σ to arrest the cell cycle, thereby gaining time for DNA repair during damage stress, which can also limit the proliferation of cancer cells.

The p21 protein plays an important role in p53‐mediated cell cycle inhibition. DNA damage or stress signals activate the p53 pathway, transcriptionally activate p21, and finally arrest the cell cycle in the G1/S phase through a series of sequential reactions.^197^, ^198^ p21 is a CDK inhibitor, a member of the KIP/CIP family, and is often regarded as a negative regulator of the cell cycle.^199^ p21 can directly inhibit DNA synthesis by competing with DNA polymerase‐δ and several other proteins involved in DNA synthesis for binding to proliferating cell nuclear antigen (PCNA) through the PCNA binding domain. p21 can also bind to and inhibit the heterodimer of CDK4/6 and cyclin D or the heterodimer of CDK2 and cyclin E through the CDK‐cyclin inhibitory domain, resulting in the inability of the downstream related cyclins to be activated, thereby arresting the cell cycle.^197^, ^200^ Downregulation of p21 is often associated with uncontrolled tumor growth and worsening symptoms.^201^, ^202^ Low p21 expression often predicts poor prognosis and more malignant cancer pathology. In NSCLC, patients with low p21 expression have lower 5‐year survival rates.^203^ In laryngeal squamous cell carcinoma, low p21 expression is associated with higher cancer pathology grade and increased lymphatic metastasis.^204^

When p53 is activated due to DNA damage, the expression of GADD45A increases, which arrests the cell cycle in the G2/M phase and facilitates DNA repair.^205^, ^206^ GADD45A is a subtype of growth arrest and DNA damage‐inducible 45 protein (GADD45), which plays an important role in cellular stress response and can be upregulated under the induction of various factors including DNA damage.^207^ GADD45 exerts the function of cell cycle control mainly by affecting the coordination between CDC2 and cyclin B and arrests the cell cycle in the G2/M period.^208^ GADD45A induces DNA demethylation during nucleotide excision repair (NER), fully exposing DNA fragments to lesion‐recognizing factors.^206^ GADD45A is downregulated in various tumors such as NSCLC, and its downregulation leads to sustained cell cycling after DNA damage, resulting in increased tumor cell heterogeneity.^209^ In oral squamous cell carcinoma (OSCC), low GADD45A expression is associated with poorer OS and predicts lower tumor cell differentiation, higher lymphatic metastasis rates, and increased cancer recurrence.^210^

p53 arrests the cell cycle in the G2/M phase by transcriptionally activating 14‐3‐3σ, and plays a role in the response of cells to DNA damage.^211^, ^212^ 14‐3‐3‐σ, also known as stratifin, is mainly found in stratified epithelial cells. 14‐3‐3‐σ, a protein encoded by the SFN gene in humans, is involved in cell cycle checkpoint control after DNA damage.^213^ Activated 14‐3‐3‐σ arrests the cell cycle in the G2/M phase by preventing the entry of the mitotic initiation complex CDC2/cyclin B1 complex into the nucleus, providing time for the repair of damaged DNA.^214^ While 14‐3‐3‐σ can be promoted by p53, it also has various abilities to regulate the level and activity of p53. 14‐3‐3‐σ can block the biological functions of ubiquitinases MDM2 and COP1, and protect p53 from being degraded by ubiquitination.^215^ In addition, 14‐3‐3‐σ can also promote the oligomerization of p53 and enhance its transcriptional activity.^216^

### 4.2 Inhibitory mechanism of p53 on angiogenesis

Angiogenesis is the development of new blood vessels from preexisting capillaries or postcapillary veins. Angiogenesis is a complex process of the coordinated action of proangiogenic and inhibitory factors.^217^ The occurrence of tumor angiogenesis, on the one hand, is due to the release of angiogenesis factors by tumor cells, which activates vascular endothelial cells, promotes the formation of new blood vessels, and provides nutrients for tumors. On the other hand, it is also due to endothelial cells stimulating the growth of tumor cells by paracrine certain angiogenic factors.^218^ p53 can inhibit angiogenesis, reduce tumor blood supply, and inhibit tumor growth and metastasis by inducing factors such as TSP‐1 and BAI1.

p53 induces the expression of thrombospondin‐1 (TSP‐1), which in turn suppresses angiogenesis by inhibiting migration and proliferation of endothelial cells.^219^ The angiogenesis inhibitor TSP‐1, which plays a crucial role in tumor growth and metastasis, ultimately alters tumor growth by altering angiogenesis in multiple tumor types.^220^ Low TSP‐1 expression in cancer promotes tumor angiogenesis.^221^

p53 can inhibit angiogenesis by overexpressing brain angiogenesis inhibitor 1 (BAI1).^222^ BAI1, a member of the G protein‐coupled receptor adhesion subfamily, was originally found to be ubiquitously expressed in brain tissue.^223^ However, some studies have shown that BAI1 is also expressed in the colon, lung, stomach, kidney, pancreas, and other tissues, and plays an important role in inhibiting angiogenesis.^222^, ^224^ Overexpression of BAI1 can inhibit tumor growth by inhibiting tumor‐induced angiogenesis.^225^, ^226^ In glioblastoma (GBM), BAI1 expression is often silenced, promoting tumor angiogenesis.^227^ Bladder transitional cell carcinoma patients with low BAI1 expression have more advanced tumor grades and increased cancer cell pleomorphism.^228^

### 4.3 p53 regulates energy metabolism in cells

Tumor cells need to consume a lot of energy to maintain growth and invasion. Unlike normal cells, the main way of energy metabolism in tumor cells is glycolysis, so tumor cells need to consume a large amount of glucose to maintain energy consumption.^229^, ^230^ In addition, the abnormal characteristics of tumor metabolism also include lipid metabolism disorders, abnormal cholesterol metabolism, abnormal protein synthesis, and so on.^231^ p53 can regulate cellular energy metabolism, inhibit glycolysis, and promote oxidative phosphorylation by inducing 5' AMP‐activated protein kinase (AMPK), tuberous sclerosis complex 2 (TSC2), PTEN, TP53‐induced glycolysis and apoptosis regulator (TIGAR), and other factors. At the same time, p53 also limits the uptake of energy substances by cancer cells, greatly inhibiting the growth of cancer cells.

Phosphorylation of p53 at Ser15 regulates metabolism by targeting and activating AMPK.^232^ AMPK is an intracellular energy sensor widely present in various eukaryotic cell lines. In humans, AMPK regulates glucose and fatty acid metabolism through multiple mechanisms.^233^ Under normal physiological conditions, activation of the AMPK signaling pathway can promote catabolism, inhibit anabolism, and increase intracellular ATP levels.^234^ When AMPK is activated by p53, it can also promote the phosphorylation and acetylation of p53, forming a positive feedback regulatory loop.^235^, ^236^ AMPK has a dual regulatory role in tumors, inhibiting cancer cell growth mainly by inhibiting glycolysis and lipid metabolism.^237^, ^238^ However, some studies have also shown that AMPK has the ability to increase the glucose intake required by tumors and is considered to be closely related to the occurrence and development of various cancers including BrC.^239^ There have been several studies targeting AMPK‐targeted therapy in cancer. For example, D‐mannose can activate AMPK, phosphorylate PD‐L1 Ser195 site, lead to abnormal glycosylation and proteasomal degradation of PD‐L1, and improve the sensitivity of triple‐negative BrC patients to immunotherapy and radiotherapy.^240^ CPI‐613 reprograms lipid metabolism through AMPK‐ACC signaling to enhance apoptosis in pancreatic cancer cells.^241^

TSC2 is transcriptionally regulated by p53, activated by AMPK, inhibits the mTOR signaling pathway, and plays a key role in regulating cellular energy metabolism.^242^, ^243^ The mammalian target of rapamycin (mTOR), a class of serine/threonine kinases, is an intracellular signaling molecule.^244^ Abnormal activation of mTOR signaling can lead to multiple adverse outcomes, including tumor formation, neonatal insulin resistance, and adipogenesis.^245^ Inactivating mutations in TSC2 cause hamartomas primarily in multiple organ systems.^246^ Loss of TSC2 enhances the sensitivity of hepatoma cell lines to everolimus and AZD8055.^247^ Low TSC2 expression leads to hyperactivation of the mTOR pathway and promotes tumor energy metabolism.^248^ TSC2 downregulation occurs in various cancers such as NSCLC, and low TSC2 expression predicts poor patient prognosis.^249^

p53 can also induce PTEN, thereby inhibiting the energy metabolism of cancer cells, and finally inhibiting tumor growth.^250^ PTEN is a protein with both protein and lipid phosphatase activities.^251^ PTEN can reduce cellular uptake of glucose and glutamine and increase mitochondrial oxidative phosphorylation by inhibiting PI3K/AKT signaling pathway. At the same time, the inhibited AKT signaling pathway can inhibit the phosphorylation and nuclear translocation of MDM2.^160^, ^161^, ^252^ By inducing PTEN, p53 limits the ubiquitination of nuclear p53 by MDM2, thereby greatly increasing the level of nuclear p53. This positive feedback regulatory loop is conducive to p53 signaling to repair damage or induce apoptosis of irreversibly damaged cells.^253^ Low PTEN expression in tumors weakens inhibition of tumor metabolism and promotes tumor development.^254^ Clinical studies have shown that low PTEN expression is a significant factor in poor cancer patient prognosis.^255^

p53 can play an important role in limiting the growth of cancer cells by inhibiting TIGAR.^256^, ^257^ TIGAR is a protein containing a functional sequence similar to the 6‐phosphofructo‐2‐kinase/fructose‐2, 6‐biphosphatase domain.^258^ TIGAR inhibits glycolysis primarily by hydrolyzing fructose‐1,6‐bisphosphate and fructose‐2,6‐bisphosphate.^259^ TIGAR also has the ability to promote the pentose phosphate pathway, which plays an important role in the process of DNA repair and ROS resistance.^260^

### 4.4 p53 promotes DNA damage repair

DNA damage repair refers to the phenomenon that DNA molecules in biological cells recover their structure after being damaged by a variety of enzymes, mainly including NER, base excision repair, mismatch repair, and DSB repair.^195^, ^261^, ^262^, ^263^, ^264^ Timely DNA repair can greatly reduce the chance of cancer.^265^ p53 can promote DNA damage repair and inhibit carcinogenesis by inducing factors such as DDB2, SESN1, and SESN2.

DNA damage‐induced high expression of p53 can activate DNA damage‐binding protein 2 (DDB2), repair DNA damage, and inhibit cancer.^266^ DDB2 is an essential protein for repairing UV‐damaged DNA, and the heterodimeric protein complex of DDB1/DDB2 is involved in NER. As a smaller subunit of this complex, DDB2 primarily functions in DNA binding.^267^ DDB2 expression is downregulated in colorectal cancer (CRC) and other cancers and is closely associated with cancer metastasis.^268^ Low DDB2 expression correlates with poor OS in stomach adenocarcinoma (STAD) patients and predicts loss of active immune response to tumors.^269^

p53 resists ROS damage by promoting the expression of SESN1,^270^ and can also prevent damage and protect cells through SESN2.^271^, ^272^ The stress‐inducible protein family is a class of conserved antioxidant proteins, which are activated when cells are under oxidative stress and protect cells from ROS through various means, including SESN1, SESN2, and SESN3. Among them, SESN1 and SESN2 are directly regulated by p53.^273^, ^274^ SESN1 activates the AMPK/PGC‐1α/UCP2 axis and inhibits mitochondrial O2‐ production by forming a complex with AMPK.^270^ SESN2 induces cells to promote DNA repair or autophagy by activating the SESN2/AMPK/TSC2 pathway.^275^

### 4.5 p53 induces apoptosis

Apoptosis plays an important role in maintaining the stability of the internal environment, and the abnormal regulation of the apoptosis pathway is closely related to the occurrence of tumors.^276^, ^277^ p53 can promote cell apoptosis and inhibit tumor development by inducing Fas, Bax, NOXA, PUMA, TP53AIP1, AIFM2, IGF‐BP3, and other factors.

DNA damage or stress can induce p53 to transcribe and activate Fas, leading to an increase in the amount of Fas on the cell membrane surface and promoting apoptosis induced by the Fas/FasL pathway.^278^, ^279^ Fas is considered an apoptotic receptor involved in Fas/FasL pathway‐induced apoptosis.^280^ FasL is activated by binding Fas and inducing its trimer formation. After activation, the Fas trimer binds to Fas‐associating via the death domain (FADD), and then FADD continues to recruit caspase 8/10.^281^ Fas trimer, FADD, and caspase 8/10 form a death‐inducing signaling complex. In this complex, precaspase 8 self‐cleaves to form active caspase 8, which initiates the downstream caspase cascade reaction and eventually leads to apoptosis.^281^, ^282^ Low Fas expression results in loss of extrinsic pathway apoptosis signaling in various tumors, leading to malignant cancer progression.^283^, ^284^ Loss of Fas expression is a major cause of poor prognosis and malignant progression in cancer patients.^285^

In addition to participating in death receptor‐mediated apoptosis, p53 can also induce mitochondria‐dependent apoptosis by upregulating the expression level of Bax.^286^, ^287^ p53 can also promote the expression of various other Bcl‐2 family proteins (such as NOXA,^288^ PUMA,^289^ etc.), and induce cell apoptosis through the mitochondrial pathway. Siva1 can transmit the apoptotic signal to the cytoplasm at the tail of CD27, a member of the tumor necrosis factor receptor family. And the ectopic expression of Siva1 can also combine with the antiapoptotic protein Bcl‐XL to promote cell apoptosis through the mitochondrial pathway.^290^ Siva1 also has the function of ubiquitinating ARF, thereby maintaining the stability of E3 ubiquitinases such as MDM2. p53 can transcriptionally activate Siva1 to play an antiapoptotic role, but at the same time, Siva1 can also induce p53 ubiquitination, forming a negative feedback regulation with p53.^291^ Low Siva1 expression enhances cancer cell resistance to apoptotic signals, induces aixib1 survival, and promotes cancer progression.^292^ Low Siva1 expression is associated with poorer OS and lower tumor differentiation in cancer patients.^293^

Phosphorylation of p53 Ser46 can transcriptionally activate tumor protein p53‐regulated apoptosis‐inducing protein 1 (TP53AIP1), thereby inducing mitochondria‐dependent apoptosis.^294^ TP53AIP1 is a protein expressed specifically in the thymus and localized to the mitochondrial membrane. TP53AIP1 induces the subsequent caspase cascade pathway to mediate apoptosis by promoting cytochrome *c* (Cyt *c*) release.^295^ Inhibited TP53AIP1 expression impairs the ability to induce apoptosis in cancer cells. Low TP53AIP1 expression in various cancers promotes cancer progression.^296^, ^297^

Apoptosis‐inducing factor mitochondria‐associated 2 (AIFM2) has significant homology to apoptosis‐inducing factor (AIF) and NADH oxidoreductase but lacks a mitochondrial localization sequence. AIFM2 is transcriptionally activated by p53, binds to DNA in a nonsequence‐specific manner, and induces caspase‐independent apoptosis.^298^ A study has also shown that AIFM2 can play an important role in the process of suppressing ferroptosis in a ubiquitin‐dependent manner.^299^

Activation of p53 signaling can upregulate the expression of insulin‐like growth factor‐binding protein 3 (IGF‐BP3), thereby promoting apoptosis.^300^ IGF‐BP3 is a growth hormone‐dependent IGF binding protein.^301^ IGF‐BP3 can bind and stabilize IGF, prevent IGF from being degraded, and promote its transportation in various parts of the body. IGF‐BP3 can also compete with IGF receptors to bind free IGF, and antagonize the proproliferation and antiapoptotic effects of IGF.^302^ IGF‐BP3 is expressed at low levels in various cancers such as cervical cancer. Low IGF‐BP3 expression inhibits cell apoptosis and promotes tumor cell proliferation.^303^ Low IGF‐BP3 expression often predicts poor patient treatment outcomes.^304^, ^305^

## 5 CLINICAL ADVANCES IN TARGETED p53 Therapy

The p53 signaling pathway plays a crucial role in cancer‐related mutations, providing numerous opportunities for therapeutic intervention.^306^ Current approaches to targeting the p53 pathway involve direct regulation of p53 and modulation of p53‐related proteins. However, as a nuclear transcription factor, p53 lacks typical drug target characteristics, presenting significant challenges in drug development and clinical application.^40^ While targeted therapy of the p53 pathway is still in its early stages, it holds immense research potential and clinical value and is expected to play a significant role in future cancer treatment.^306^

### 5.1 The p53‐targeted drugs

Drugs that target p53 directly work to restore or boost its tumor‐suppressing abilities through a variety of methods such as restoring its conformation, increasing its transcriptional activity, improving its structural stability, and preventing its degradation. Given that p53 mutations are present in over half of all cancers, targeting mutated p53 represents a promising approach. However, research in this area is still in its early stages, with most drugs in preclinical trials and only a few in clinical trials or actual clinical applications (Table 1). These include arsenic trioxide (ATO) and COTI‐2 targeting R175H; APR‐246 and APR‐548 targeting R273H; atorvastatin targeting R280K; phenethyl isothiocyanate (PEITC) targeting both R273H and R280K; and PC14586 targeting Y220C. Several other p53‐targeted drugs are still under development, including PhiKan5196,^307^ L5,^308^ and PK9318^309^ targeting Y220C; ADH‐6 targeting R248W^310^; and Peptide 46 targeting R273H.^311^ ATO was found to promote leukocyte apoptosis through multiple mechanisms, including targeting mutant p53, and has been applied in the treatment of acute promyelocytic leukemia.^312^ COTI‐2 selectively recognizes and restores mutant p53 through structure‐based design.^313^, ^314^ APR‐246 is a potential anticancer drug developed by Aprea that restores and activates inactive p53 protein to regain its normal tumor suppressor function. Its precursor, PRIMA, was first identified in 2002 from a library of low‐molecular‐weight compounds targeting p53 mutations.^315^ APR‐246 has shown potential therapeutic effects in clinical trials, particularly for tumors harboring p53 mutations.^316^, ^317^ APR‐548, a new derivative of APR‐246 developed by Aprea, has stronger cell membrane penetration and is believed to have greater anticancer activity.^40^ Atorvastatin is a statin drug that lowers cholesterol and lipid levels by inhibiting the enzyme 3‐hydroxy‐3‐methylglutaryl‐coenzyme A reductase to reduce cholesterol synthesis in the body. However, recent studies have shown that Atorvastatin can also indirectly exert a tumor‐suppressing effect by affecting missense p53.^318^, ^319^ PEITC is a natural compound extracted from cruciferous plants with broad anticancer properties.^320^, ^321^ Recent research indicates that PEITC targets multiple mutations such as p53‐R273H and p53‐R280K and reverses the ability of p53 mutant cancer cells.^322^ PC14586, an orthotic of p53‐Y220C, has been shown to be tolerable and effective in clinical trials.^323^ However, due to the complex dysregulated environment in cancer, it is often difficult to achieve optimal therapeutic effects by targeting p53 alone.^324^, ^325^ As such, combining p53‐targeted drugs with other commonly used broad‐spectrum anticancer drugs represents a promising approach to cancer treatment. Several p53‐targeted drugs have been combined with common anticancer drugs and entered clinical trials, including acalabrutinib, azacitidine, carboplatin, DDP, cytarabine, dabrafenib, decitabine, pembrolizumab, pegylated liposomal DOX hydrochloride (PLD), and venetoclax.

**TABLE 1:** Drugs targeting the p53 signaling pathway are currently undergoing clinical trials.

Target		Targeted drug	Combination therapy	Indication	Phase	Trial ID
mut‐p53	p53‐R175H	ATO	—	OC and EC	NA	NCT04489706^353^
		ATO	—	Multiple tumors	II	NCT04695223^353^
		ATO	—	Solid tumors	II	NCT04869475^353^
		ATO	Cytarabine/decitabine	AML	II	NCT03381781
		ATO	Decitabine	MDS	III	NCT03377725
		COTI‐2	—	Malignancies	I	NCT02433626
		COTI‐2	Cisplatin	Malignancies	I	NCT02433626
	p53‐R273H	APR‐246	—	Hematologic neoplasms and PRAD	I	NCT00900614
		APR‐246	—	AML and MDS	II	NCT03931291^354^
		APR‐246	—	ESCA	I/II	NCT02999893
		APR‐246	Acalabrutinib	Lymphoma	I/II	NCT04419389
		APR‐246	Azacitidine	AML, MPN, and CMML	I/II	NCT03588078^355^
		APR‐246	Azacitidine	AML, MPN, and CMML	I/II	NCT03072043^356^
		APR‐246	Carboplatin/PLD	OC	I/II	NCT02098343
		APR‐246	Dabrafenib	Melanoma	I/II	NCT03391050
		APR‐246	Pembrolizumab	Solid tumors	I/II	NCT04383938^357^
		APR‐246	PLD	OC	II	NCT03268382
		APR‐246	Venetoclax	Lymphoma	II	NCT04990778
		APR‐246	Venetoclax	Lymphoma	I/II	NCT04419389
		APR‐246	Venetoclax/azacitidine	AML	I	NCT04214860^358^
		APR‐548	Azacitidine	MDS	I	NCT04638309
	p53‐R280K	Atorvastatin	—	CRC	II	NCT04767984
		Atorvastatin	—	AML	I	NCT03560882
	p53‐R280K and R273H	PEITC	—	Oral cancer	I/II	NCT01790204^359^
	p53‐Y220C	PC14586	—	Solid tumors	I/II	NCT04585750
p53‐related protein	MDM2	ALRN‐6924	Cytarabine	Leukemia, brain tumor, solid tumor, and lymphoma	I	NCT03654716
		Alrizomadlin	Azacitidine/cytarabine	AML and MDS	I	NCT04275518
		Ivaltinostat	—	Solid tumors	I	NCT01226407
		Idasanutlin	Cytarabine/daunorubicin	AML	I/II	NCT02407080
		Milademetan	—	AML and MDS	I	NCT02319369
		Navtemadlin	Cytarabine/idarubicin hydrochloride	AML	I	NCT04190550
		NVP‐CGM097	—	Solid tumors	I	NCT01760525^337^
		RO5045337	—	Neoplasms, and myelogenous leukemia	I	NCT01677780
		RO6839921	—	Neoplasms, and myelogenous leukemia	I	NCT02098967^360^
		SAR405838	—	Neoplasm malignant	I	NCT01636479^361^
		Siremadlin	LEE011	Liposarcoma	I	NCT03760445
	AMPK	Acadesine	—	Leukemia	I/II	NCT00559624
		ASP4132	—	Lymphoma, and solid tumors	I	NCT02383368^340^
		HL271	—	Solid tumors	I	NCT03272256
		Metformin	—	BrC	II	NCT01266486^362^
		Metformin	—	EC	I	NCT01911247^363^
		Metformin hydrochloride	—	GBM	II	NCT04945148
		Phenformin hydrochloride	Trametinib/dabrafenib	Melanoma	I	NCT03026517^364^

ATO, arsenic trioxide; AML, acute myeloid leukemia; BrC, breast cancer; CMML, chronic myelomonocytic leukemia; CRC, colorectal cancer; EC, endometrial cancer; ESCA, esophageal carcinoma; GBM, glioblastoma; MDS, myelodysplastic syndromes; MPN, myeloproliferative neoplasm; OC, ovarian cancer; PEITC, phenethyl isothiocyanate; PLD, pegylated liposomal doxorubicin; PRAD, prostate adenocarcinoma.

The clinical trial data are sourced from clinicaltrials.gov

### 5.2 Drugs targeting p53 signaling‐related proteins

Targeting proteins related to p53 signaling is another viable approach to p53 signaling targeted therapy. This can be achieved through upstream activation of p53 signaling or downstream blocking of abnormal p53 signaling. However, currently only drugs targeting MDM2/MDMX and the downstream metabolic regulatory protein AMPK have entered clinical trials (Table 1). These include ALRN‐6924, alrizomadlin, ivaltinostat, idasanutlin, milademetan, navtemadlin, NVP‐CGM097, RO5045337, RO6839921, SAR405838, and siremadlin targeting MDM2/MDMX; and acadesine, ASP4132, HL271, metformin, metformin hydrochloride, and phenformin hydrochloride targeting AMPK.

MDM2/MDMX is a molecular chaperone of p53 that can inhibit its effect through ubiquitination. Targeting MDM2/MDMX is therefore an effective way to enhance p53's anticancer activity. The mechanisms of action of MDM2/MDMX‐targeted drugs currently in clinical trials include inhibiting the interaction between MDM2 and p53, inhibiting MDM2 ubiquitin ligase activity, and downregulating MDM2 expression levels.^326^ ALRN‐6924, developed by Aileron Therapeutics, is a peptide inhibitor with dual activity targeting MDM2 and MDMX. It inhibits the interaction between MDM2/MDMX and p53 protein.^327^ RG7112, developed by Roche Pharmaceuticals, inhibits the combination of MDM2 and p53 by binding to the p53 binding site on MDM2.^328^ RO6839921, developed by Genentech Biotechnology, is an intravenous prodrug of idasanutlin,^329^ which selectively antagonizes MDM2 and inhibits its binding to p53.^330^ Ivaltinostat indirectly increases MDM2 levels by promoting p53 expression.^331^ Milademetan disrupts the interaction between MDM2 and p53.^332^ Alrizomadlin inhibits the MDM2–p53 interaction by binding to MDM2.^333^ Navtemadlin also inhibits the MDM2–p53 interaction.^334^ SAR405838 is a potent and selective inhibitor of the MDM2–p53 interaction.^335^ Siremadlin selectively inhibits the p53–MDM2 interaction.^336^ NVP‐CGM097 potently and selectively inhibits HDM2, the human homolog of MDM2, and its interaction with p53.^337^

AMPK mediates the regulation of metabolism by p53 signaling and inhibits glycolysis and fat metabolism to suppress tumors. Targeting AMPK therefore represents a promising approach to therapy. The mechanisms of action of AMPK‐targeted drugs currently in clinical trials include inhibiting mitochondrial respiration to increase the AMP‐to‐ATP ratio and acting as an AMP analog to indirectly activate AMPK. Acadesine is an adenosine analog that activates AMPK.^338^ ASP41322 is a novel mitochondrial complex I inhibitor that increases the AMP‐to‐ATP ratio and indirectly activates AMPK.^339^, ^340^ Metformin inhibits the mitochondrial respiratory chain in the liver, leading to AMPK activation by increasing the AMP‐to‐ATP ratio.^341^ Metformin hydrochloride, phenformin hydrochloride, and HL271 are all biguanide antidiabetic drugs with similar effects to metformin and can activate AMPK.^341^, ^342^, ^343^ However, given AMPK's dual regulatory role in tumors, the value of AMPK‐targeted drugs in cancer requires further exploration through clinical data.

Several other drugs targeting p53 signaling‐related proteins are still under development, including MD‐222,^344^ Navtemadlin‐d7,^345^ and MX69^346^ targeting MDM2; ASP4132,^346^ BAY‐3827,^347^ and Aldometanib^348^ targeting AMPK^339^; bpV(phen),^349^ VO‐OHPic,^350^ and Coelonin^351^ targeting PTEN; and iFSP1^352^ targeting AIFM2.

## 6 ONCOGENE TRIAP1: A NOVEL TARGET DOWNSTREAM OF p53

p53 often promotes apoptosis in cells, while its downstream target TRIAP1 mediates its antiapoptotic effect. TRIAP1 reduces mitochondrial membrane permeability, inhibits mitochondrial‐dependent apoptotic pathways, and counteracts the effects of other p53‐related proapoptotic proteins.^365^ The transcription of TRIAP1 is activated by p53 signaling, which may contribute to the failure of p53‐targeted therapy.

**FIGURE 3:**
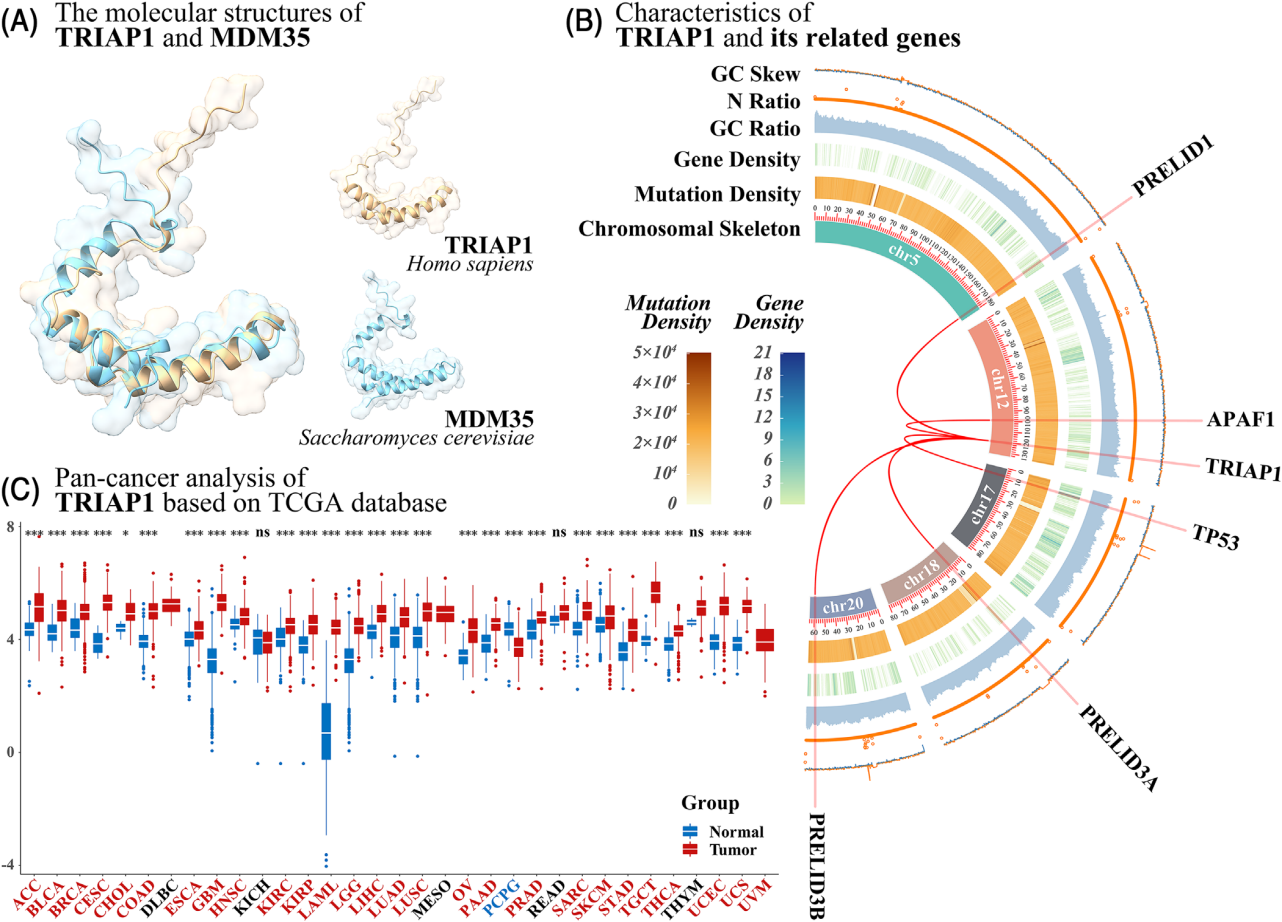
The structure, related genes, and a pan‐cancer analysis of TRIAP1. (A) Molecular structure comparison of human protein TRIAP1 (gold) and yeast protein MDM35 (cyan). TRIAP1 and MDM35 have similar protein structures, but the N‐terminus of MDM35 has one more short α‐helix structure than TRIAP1, and the C‐terminus is more flexible than TRIAP1. (B) The sequence characteristics of TRIAP1 and its related genes in chromosomes. (C) A pan‐cancer analysis of TRIAP1 based on TCGA database. *** means adjusted *p* < 0.001; ** means adjusted *p* < 0.01; * means adjusted *p* < 0.05; ns means no significant difference. TRIAP1 was significantly upregulated (adjusted *p* < 0.05) in 26 tumors, including ACC, BLCA, BRCA, CESC, CHOL, COAD, ESCA, GBM, HNSC, KIRC, KIRP, LAML, LGG, LIHC, LUAD, LUSC, OV, PAAD, PRAD, SARC, SKCM, STAD, TGCT, THCA, THYM, UCEC, and UCS. TRIAP1 was significantly downregulated in PCPG (adjusted *p* < 0.05). TRIAP1 was not significantly changed in three tumors (KICH, READ, and THYM). There were no controls in three tumors (DLBC, MESO, and UVM). Please check GDC (https://gdc.cancer.gov/resources‐tcga‐users/tcga‐code‐tables/tcga‐study‐abbreviations) for the full name of the TCGA abbreviations.

**FIGURE 4:**
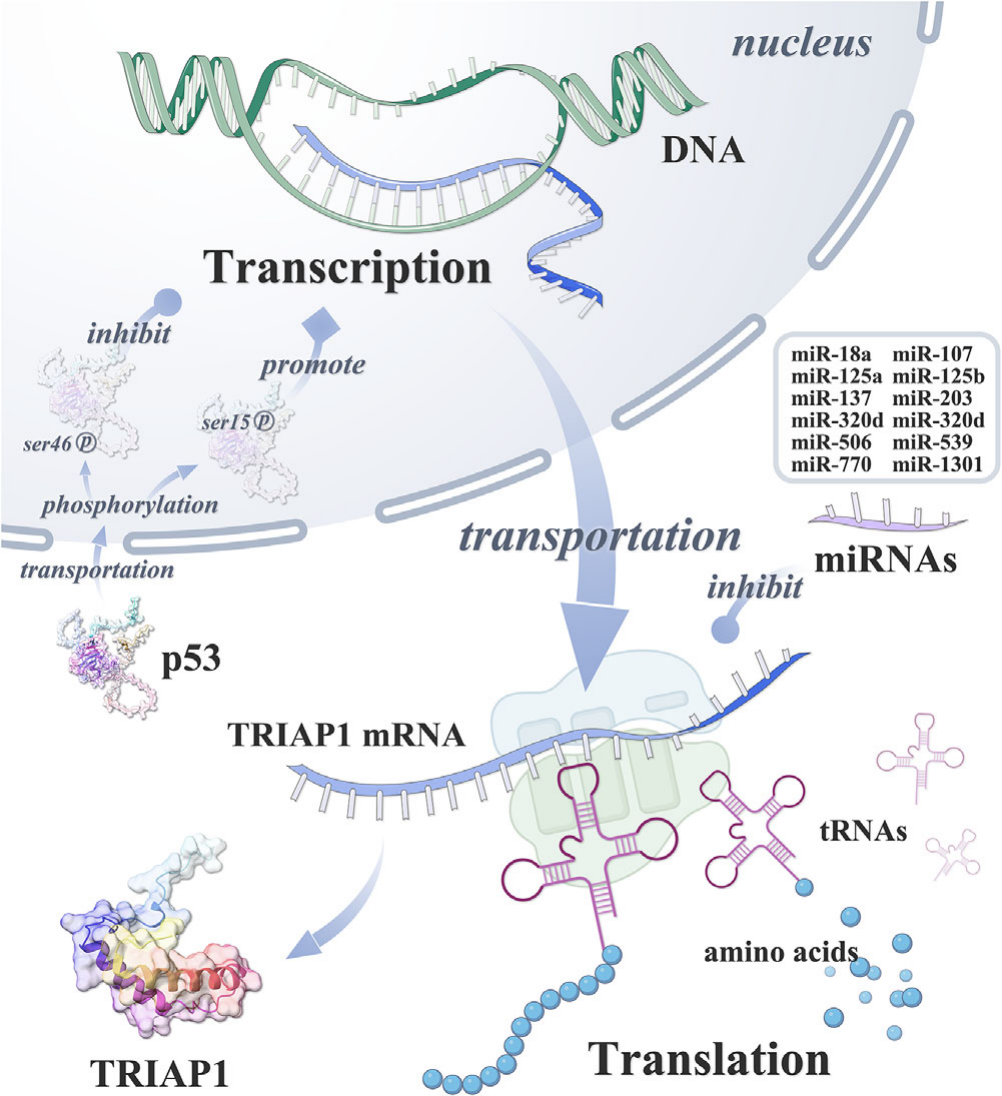
The regulatory factors of TRIAP1 expression. TRIAP1 is regulated in different stages during the expression process, including p53 protein regulating TRIAP1 at the transcriptional stage, and 12 miRNAs targeting TRIAP1 at the translational stage.

TRIAP1, also known as a p53‐inducible cell‐survival factor (p53CSV), is an evolutionarily conserved antiapoptotic protein.^366^ TRIAP1 is localized to the minus strand of 12q24.31, consists of two exons and one intron (Figures 3 and 4), and can be transcribed into two transcripts. Among them, the transcript TRIAP1‐202 (1151 bp) can be translated into protein, while the transcript TRIAP1‐201 (1153 bp) does not contain an open reading frame.

TRIAP1 is a homolog of MDM35, the sequence and structure of the two are similar, but the N‐terminus of MDM35 also has a short α‐helix, and the C‐terminus is more flexible than TRIAP1 (Figure 3).^367^ Similar to MDM35,^368^ TRIAP1 can form a heterodimer with protein of relevant evolutionary and lymphoid interest (PRELI) protein and play the role of transporting phospholipids in the mitochondrial intermembrane space.^369^ TRIAP1 can inhibit mitochondrial cleavage and the activation of downstream apoptotic protease activating factor‐1 (APAF1)/apoptosome, and play an antiapoptotic effect.^370^ Experiments have shown that the expression level of TRIAP1 is generally elevated in 9 types of cancers (Table 2).

**TABLE 2:** The upregulation and effect of TRIAP1 in human cancers.

System	Cancer	Level	Expression	Normal group	Tumor group	Effect in vitro	Cell line	Effect in vivo	Animal model	References
Blood system	MM	Tissue	Upregulated	3 pools of normal plasma cells from palatine tonsils	CD138^+^ MM cell from 31 newly diagnosed MM patients	—	—	—	—	^371^
		Cell	Upregulated	Reactive tonsils plasma cells	RPMi8226 and U266	Apoptosis↓	RPMI8226	—	—	^372^
Digestive system	GC	Tissue	Upregulated	Para‐carcinoma tissues from 12 GC patients	Tumor tissues from 12 GC patients	Proliferation↑, invasion↑, migration↑, and apoptosis↓	NCI‐N87	Growth↑	NCI‐N87 cell xenograft in BALB/c athymic nude mice	^43^
	HCC	Cell	Upregulated	HL‐7702	PLC/PRF/5	Migration↑, viability↑, and apoptosis↓	PLC/PRF/5	—	—	^373^
	OSCC	—	—	—	—	Proliferation↑ and migration↑	CAL‐27 and OSC‐4	—	—	^374^
Endocrine system	TC	Tissue	Upregulated	Para‐carcinoma tissues from 43 TC patients	Tumor tissues from 43 TC patients	Proliferation↑, invasion↑, migration↑, and apoptosis↓	TPC‐1 and K1	—	—	^375^
Motor system	SaOS	Tissue	Upregulated	Normal bone tissues from 45 healthy volunteers	Tumor tissues from 52 SaOS patients	Apoptosis↓ and DOX‐resistance↑	KHOS and U2OS	Growth↑	KHOS cell xenograft in BALB/c nude mice	^376^
		—	—	—	—	Proliferation↑ and apoptosis↓	U2OS and SAOS‐2	—	—	^377^
		—	—	—	—	Proliferation↑, invasion↑, migration↑, and apoptosis↓	143b and MG63	Growth↑ and metastasis↑	143b cell xenograft in BALB/nude mice	^370^
Reproductive system	BrC	Cell	Upregulated	HMEC‐TERT and 226‐L‐U19	CAL51, MDA‐MB‐231, T47D and ZR75	DOX‐resistance↑	CAL51 and MCF7	—	—	^378^
		—	—	—	—	Viability↑, apoptosis↓, and PTX‐resistance↑	MCF‐7	—	—	^379^
	OC	—	—	—	—	DDP‐resistance↑ and apoptosis↓	SKOV3	Growth↑ and DDP‐resistance↑	SKOV3 cell xenograft in BALB/c nude mice	^44^
		—	—	—	—	Cell cycle↑, proliferation↑, and apoptosis↓	A2780s and A2780cp	Growth↑	A2780cp cell xenograft in BALB/c nude mice	^380^
	PCa	—	—	—	—	Proliferation↑, invasion↑, and migration↑	PC‐3 and C4‐2B	Growth↑	PC‐3 cell xenograft in BALB/c nude mice	^381^
		—	—	—	—	Apoptosis↓ and TKI‐resistance↑	RasB1	Growth↑ and metastasis↑	RasB1 cell xenograft in male nude mice	^382^
		—	—	—	—	Apoptosis↓ and radiation‐resistance↑	PC3 and LNCaP	Growth↑ and radiation‐resistance↑	PC3 and WMPY‐1 cell xenograft in NMRI nude mice	^383^
	PeCa	Tissue	Upregulated	Para‐carcinoma tissues from 15 NSCLC patients	tumor tissues from 57 PeCa patients	—	—	—	—	^384^
Respiratory system	NPC	Tissue	Upregulated	8 normal nasopharyngeal epithelium specimens	tumor tissues from 16 NPC patients	Proliferation↑ and apoptosis↓	CNE‐2 and SUNE‐1	Growth↑	SUNE‐1 cell xenograft in BALB/c nude mice	^385^
		Cell	Upregulated	NP69	CNE‐2, SUNE‐1, CNE‐1, HNE‐1, HONE‐1 and C666‐1					
	NSCLC	Tissue	Upregulated	Para‐carcinoma tissues from 45 NSCLC patients	tumor tissues from 45 NSCLC patients	Proliferation↑ and apoptosis↓	A549	—	—	^386^
		—	—	—	—	Ionizing radiation‐resistance↑	A549 and H460	—	—	^366^

“↑” means that the biological behavior is promoted, “↓” means that the biological behavior is inhibited. BrC, breast cancer; EOC, epithelial ovarian cancer; GC, gastric cancer; HCC, hepatocellular carcinoma; MM, multiple myeloma; NPC, nasopharyngeal carcinoma; NSCLC, non‐small‐cell lung cancer; OC, ovarian cancer; OSCC, oral squamous cell carcinoma; PCa, prostate cancer; PeCa, penile carcinoma; SaOS, osteosarcoma; TC, thyroid cancer; PTX, taxol; DOX, doxorubicin; DDP, cisplatin; TKI, tyrosine kinase inhibitor.

### 6.1 The molecular mechanism of TRIAP1

**FIGURE 5:**
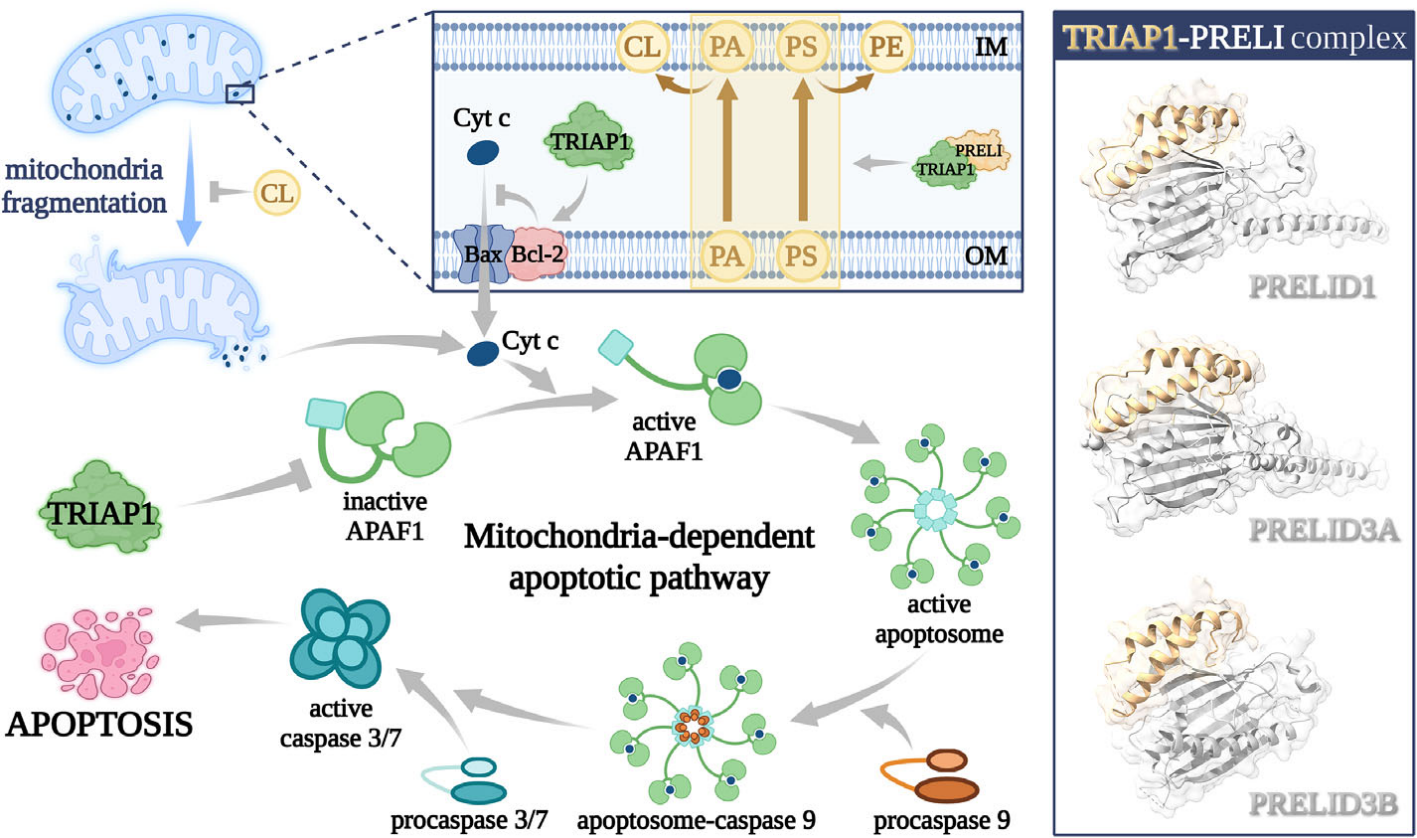
TRIAP1 inhibits mitochondria‐dependent apoptosis. TRIAP1 exerts antiapoptotic effects by inhibiting mitochondria‐dependent apoptosis pathways through various regulatory effects. TRIAP1 can ensure CL accumulation by promoting phospholipid transport in the mitochondrial intermembrane space and inhibiting mitochondrial lysis; TRIAP1 can also directly interact with APAF1 and inhibit the activation of apoptosomes. Cyt *c*, cytochrome *c*; PA, phosphatidic acid; PS, phosphatidylserine; PE, phosphatidylethanolamine; CL, cardiolipin; OM, outer membrane; IM, inner membrane.

As shown in Figure 4, The expression of TRIAP1 is regulated by multiple mechanisms, including the regulation of TRIAP1 transcription by p53 protein^41^ and the targeted repression of TRIAP1 by 12 microRNAs (miRNAs). As shown in Figure 5, TRIAP1 can form a heterodimeric complex with PRELI‐like family proteins and inhibit mitochondrial lysis, inhibit the activation of apoptotic peptidase activating factor 1 (APAF1)/apoptosome, and then inhibit apoptosis.^41^

A functional p53‐binding sequence (p53BS) is present in exon 1 of TRIAP1 (Figure S1). TRIAP1 expression is dependent on p53 protein, and it is hardly expressed in p53^−/−^ HCT116 cells. Phosphorylation modifications at different sites of p53 protein also have different effects on the expression of TRIAP1.^41^ Among them, p53 Ser15 phosphorylation can be induced when DNA is slightly damaged, thereby promoting the expression of TRIAP1, which in turn induces cell survival and damaged DNA repair. When DNA is severely damaged, p53 Ser46 phosphorylation can be induced, which will reduce the expression level of TRIAP1 and induce apoptosis.^41^ The expression of TRIAP1 is regulated by a variety of ncRNAs at the posttranscriptional level, including miRNAs, circular RNAs (circRNAs), and long noncoding RNAs (lncRNAs) (Table S1). miRNA can bind to the 3'‐UTR of target gene messenger RNA (mRNA), thereby inducing the degradation of target mRNA or inhibiting its expression.^387^ Competing endogenous RNAs (ceRNAs), including lncRNAs and circRNAs, can act as molecular sponges for miRNAs and competitively inhibit the targeting of miRNAs to protein‐coding genes.^388^ To date, 12 miRNAs have been reported to target TRIAP1, including miR‐18a in epithelial ovarian cancer,^380^ miR‐107 in BrC,^379^ gastric cancer (GC),^43^ NSCLC,^386^ and OSCC,^374^ miR‐125a‐5p in HCC^373^ and thyroid cancer (TC),^375^ miR‐137 in osteosarcoma (SaOS),^376^ miR‐203 in PCa,^382^ miR‐320b in nasopharyngeal carcinoma (NPC),^385^ miR‐506 in PCa,^381^ miR‐539 in SaOS,^370^ and miR‐1301 in SaOS,^377^ and miR‐125b‐5p,^389^ miR‐320d,^390^ and miR‐770‐5p^391^, ^392^ in noncancerous disease. Three of these miRNAs are also involved in ceRNA axes, including the circPVT1/miR‐137/TRIAP1 in SaOS,^376^ MFI2‐AS1/miR‐125a‐5p/TRIAP1 in TC,^375^ and PCGEM1/miR‐506/TRIAP1 in PCa.^381^ Bioinformatics analysis revealed a strong correlation between the expression of TRIAP1 and TP53 gene mutation and RNA m6A modification (see Supplementary Materials).

PRELI family proteins can form a mitochondrial intermembrane complex with TRIAP1.^393^, ^394^ Three proteins in the PRELI family (including PRELID1, PRELID3A, and PRELID3B) have PRELI‐like domains, which are involved in the regulation of phospholipid transport in mitochondria,^395^ and are closely related to a variety of malignant tumors.^396^ Phosphatidic acid (PA), the precursor phospholipid of cardiolipin (CL), is transferred to the inner mitochondrial membrane by the TRIAP1/PRELI complex and converted to CL by CL synthase.^397^ Deletion of TRIAP1 or PRELI reduces the CL content in the mitochondrial membrane, which in turn promotes mitochondrial lysis and Cyt *c* release, ultimately leading to apoptosis.^365^ TRIAP1 can inhibit the expression of the proapoptotic protein Bax, or interact with APAF1 and HSP70, inhibit the formation of downstream apoptosomes, and reduce the level of caspase activation, ultimately inhibiting apoptosis.^392^ Bcl‐2 family‐related proteins are a class of apoptosis regulators, among which Bax overexpression promotes the release of Cyt *c* from mitochondria. When Bcl‐2 is overexpressed, it can form a heterodimer with Bax and inhibit the effect of Bax on promoting the release of Cyt *c* from mitochondria.^398^ TRIAP1 can increase the level of Bcl‐2 and inhibit the level of Bax in cells, thereby inhibiting the release of Cyt *c* in mitochondria, inhibiting the activation of AFAF1/apoptosome, and inducing cell survival.^392^ Heat shock protein (HSP) is a large protein with a highly conserved sequence involved in the folding and maturation of other proteins.^399^ APAF1/apoptosome plays a key role in embryonic development and mitochondria‐dependent apoptosis.^400^ Among them, HSP70 can bind to APAF1 and inhibit its activity, while TRIAP1 can interact with HSP70 and/or APAF1, thereby inhibiting the activation of APAF1,^41^ and inhibiting the activation of the apoptosome, reducing caspase activity and inducing cell survival.^372^, ^389^

### 6.2 The dysregulated TRIAP in cancer

The inhibition of the apoptosis pathway is an important reason for the occurrence and development of cancer.^401^ As shown in Table 2, many studies have so far confirmed that TRIAP1 is highly expressed in cancers of various human systems and plays a role in promoting cancer. When TRIAP1 expression is inhibited, the malignant phenotype of cancer cells is also inhibited.

In the blood system, multiple myeloma (MM) is characterized by the transformation of plasma cells in the bone marrow into cancer cells and clonal proliferation.^402^ TRIAP1 is highly expressed in MM,^371^ and overexpressed TRIAP1 can inhibit the activation of APAF1/apoptosome, thereby inhibiting cancer cell apoptosis, leading to cancer progression.^372^

In the digestive system, GC is cancer that occurs in the mucosa of the stomach.^403^ Overexpressed miR‐107 inhibited the proliferation, invasion, and metastasis of GC cell line (NCI‐N87) by targeting TRIAP1, and promoted apoptosis. Similarly, low expression of TRIAP1 inhibited tumor progression in NCI‐N87 cell xenograft in BALB/c athymic nude mice.^43^ HCC is the most common type of chronic liver cancer in adults.^404^ Overexpressed miR‐125a‐5p targeting TRIAP1 downregulates the viability of HCC cell line PLC/PRF/5, inhibits cell invasion and migration, and promotes apoptosis.^373^ OSCC is a type of cancer that produces lesions in the histiocytic and squamous cells of the oral cavity.^405^ In OSCC, the highly expressed miR‐107 targeted the inhibition of TRIAP1, which in turn inhibited the proliferation, invasion, and migration of OSCC cell lines (CAL‐27 and OSC‐4).^374^

In endocrine system neoplasm, TC is a malignant tumor of the thyroid tissue that occurs in the Endocrine system.^406^ In TC, the highly expressed MFI2‐AS1 upregulated the expression level of TRIAP1 by competitively inhibiting miR‐125a‐5p, inhibited the apoptosis of cancer cells, and promoted the proliferation, invasion, and metastasis of cancer cells.^375^

In the motor system, SaOS is a malignant tumor that can arise in all bone tissues and is characterized by rapid development and early metastasis.^407^ In SaOS, highly expressed circPVT1 upregulates the expression level of TRIAP1 by competitively inhibiting miR‐137 and hinders the apoptosis of cancer cells (KHOS and U2OS), ultimately promoting the malignant progression of cancer.^376^ Meanwhile, the circPVT1/miR‐137/TRIAP1 axis promoted tumor growth in xenografted BALB/c nude mice.^376^ Overexpressed miR‐539^370^ and miR‐1301^377^ can suppress the malignant phenotype of SaOS cell lines and promote cancer cell apoptosis by targeting TRIAP1. Meanwhile, low expression of TRIAP1 can also inhibit tumor growth and metastasis in xenografted BALB/nude mice.^370^

In the reproductive system, BrC is cancer derived from breast tissue.^408^ In BrC, highly expressed miR‐107 targets TRIAP1 in the BrC cell line (MCF‐7), leading to down‐regulation of cancer cell MCF‐7 viability and promoting cancer cell apoptosis.^379^ Overexpressed TRIAP1 also blocked the activation of APAF1/apoptosome and promoted the malignant phenotype of BrC cell lines (CAL51 and MCF7).^378^ Ovarian cancer (OC) is cancer that originates in the ovaries of women.^409^ In OC, overexpressed TRIAP1 inhibits apoptosis of cancer cell SKOV3 by inhibiting the activation of APAF1/apoptosome.^44^ Overexpressed miR‐18a targets and inhibits TRIAP1, thereby inhibiting the cell cycle and proliferation of cancer cells (A2780cp and A2780s) and promoting cancer cell apoptosis. Meanwhile, low expression of TRIAP1 inhibited tumor growth in xenografted BALB/c nude mice.^380^ PCa is a malignant tumor originating from the male prostate.^410^ In PCa, overexpressed PCGEM1 competitively represses miR‐506, which in turn leads to the upregulation of TRIAP1.^381^ In PCa, overexpressed PCGEM1 or TRIAP1 can inhibit apoptosis and promote the malignant phenotype of cancer cells (PC‐3 and C4‐2B)^383^ and promote tumor growth in xenografted BALB/c nude mice.^381^ Overexpressed miR‐203 can target and inhibit TRIAP1, thereby promoting apoptosis of cancer cells (RasB1). Meanwhile, low expression of TRIAP1 inhibited tumor growth and metastasis in xenografted male nude mice.^382^

In the respiratory system, NPC is cancer that develops in the upper throat or nasopharyngeal cavity.^411^ In NPC, overexpressed miR‐320b targets and inhibits TRIAP1, promotes mitochondrial fragmentation and Cyt *c* release, which in turn inhibits the proliferation of cancer cells (CNE‐2 and SUNE‐1) and promotes cancer cell apoptosis.^385^ Meanwhile, low expression of TRIAP1 inhibits tumorigenesis in xenografted BALB/c nude mice.^385^ NSCLC is any type of lung cancer of epithelial origin other than small‐cell lung carcinoma.^412^ In NSCLC, overexpressed miR‐107 targets TRIAP1, which in turn inhibits proliferation and promotes apoptosis in an NSCLC cell line (A549).^386^

### 6.3 Prognostic value of TRIAP1

In NPC, NSCLC, and penile carcinoma (PeCa), overexpressed TRIAP1 is a high‐risk factor for patient survival. Among them, patients with NPC and NSCLC overexpressing TRAIP1 had shorter OS^366^, ^385^ and DFS.^385^ PeCa patients overexpressing TRIAP1 have higher tumor histological grades and shorter local RFS and thus can be used as a predictor of cancer recurrence.^384^

In TCGA data, high expression of TRIAP1 was a high‐risk factor for cancer patient survival, and it was significantly associated with poorer OS in 7 tumors and poorer disease‐specific survival (DSS) in eight tumors (hazard ratio (HR) > 1 and *p* < 0.05). This is consistent with the results mentioned above that the expression of TRIAP1 in these cancer tissues is higher than that in the corresponding normal tissues, indicating that TRIAP1 can be used as a marker for the diagnosis and prognosis of these cancers. (See Supplementary Materials and Figure S2A for details.)

We also calculated the correlation of TRIAP1 with the tumor microenvironment and tumor stemness. The results also showed that in most cancers, TRIAP1 was negatively correlated with ESTIMATE score^413^ and positively correlated with cancer‐associated fibroblast (CAF) hallmarks^414^ and tumor stemness^415^ (see Supplementary Materials and Figures S2B and S2C). Low activity of antitumor immune cells, high activity of CAFs, and high tumor stemness may be the reasons for malignant tumor progression and poor prognosis in patients with high TRIAP1 expression.

### 6.4 Potential cancer therapy targeting TRIAP1

**FIGURE 6:**
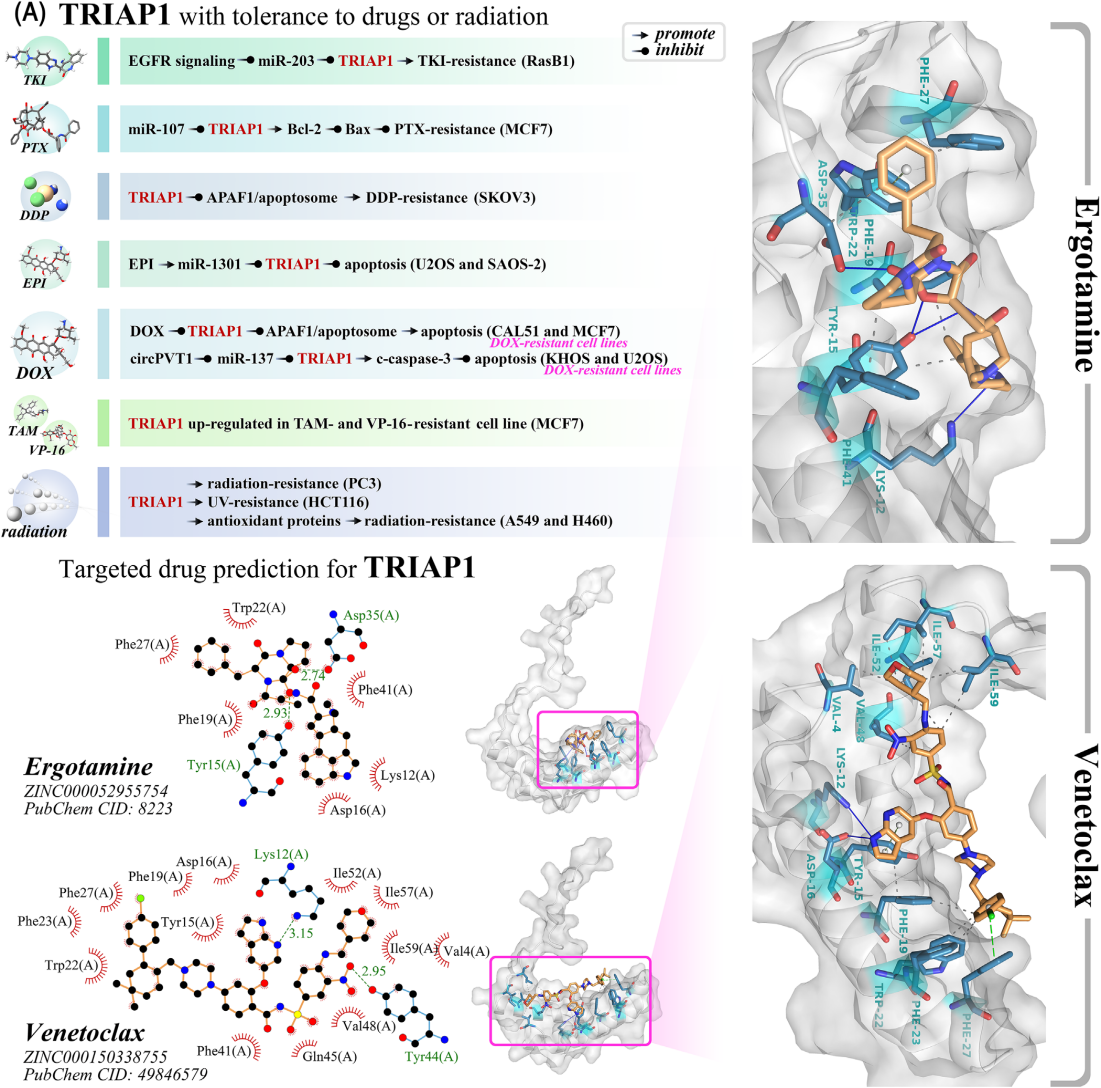
The potential role of TRIAP1 in chemotherapy and radiotherapy. (A) TRIAP1 can promote the resistance of cancer cells to radiation and various anticancer drugs, including DOX, DDP, TKI, TAM, and PTX. PTX, taxol; DDP, cisplatin; EPI, epirubicin; DOX, doxorubicin; TAM, tamoxifen; VP‐16, etoposide. (B) Interaction of Ergotamine and Venetoclax with protein production. The protein backbone is shown in silver gray, the ligands are shown in orange, and the active site amino acids are shown in cyan. In the 2D diagram, the green dotted line shows the hydrogen bonds and their bond lengths, and the red arcs and black atoms represent the residues and atoms involved in hydrophobic interactions, respectively. In the 3D diagram, the gray dashed lines represent hydrophobic interactions, the blue solid lines represent hydrogen bonds, and the green dashed lines represent π–π stacking bonds. Ergotamine has a hydrogen bond interaction with Tyr15, Phe19, Trp22, Phe27, Asp35, and Phe41 of TRIAP1, a hydrophobic interaction with Lys12, Tyr15, Asp35, Phe19, Phe27, Trp22, Phe41, and Asp16 of TRIAP1, and forms a stacking bond with Trp22 of TRIAP1. Venetoclax has hydrogen bond interactions with Lys12, Asp16, and Tyr44 of TRIAP1. Venetoclax forms hydrophobic interactions with Val4, Tyr15, Asp16, Phe19, Trp22, Phe23, Phe27, Phe41, Gln45, Val48, Ile52, Ile57, and Ile59 of TRIAP1. Venetoclax forms π–π stacking bonds with Tyr15 and Phe27 of TRIAP1.

The drug resistance of tumor cells is one of the important reasons for the failure of chemotherapy and radiotherapy. TRIAP1 plays an important role in promoting tumor resistance to treatment. TRIAP1 inhibits cell apoptosis by blocking the mitochondria‐dependent apoptotic pathways and induces tolerance. As shown in Table S2, TRIAP1 is more expressed in multidrug‐resistant cancer cell lines (DOX, DDP, TAM, and VP‐16) than in corresponding sensitive cell lines. As shown in Figure 6A, TRIAP1 can promote the resistance of cancer cells to radiation and various anticancer drugs, including DOX, DDP, tyrosine kinase inhibitor (TKI), TAM, and taxol (PTX).

DOX is an antitumor antibiotic, which can inhibit the synthesis of RNA and DNA. DOX has a broad antitumor spectrum and has an effect on a variety of tumors, and has a killing effect on tumor cells in various growth cycles.^416^ In BrC^378^ and SaOS,^376^ TRIAP1 was significantly upregulated in DOX‐resistant cell lines than in corresponding DOX‐sensitive cell lines. Low and moderate doses of DOX promote TRIAP1 expression and induce DOX resistance in BrC cell lines (CAL51 and MCF7).^378^ However, high doses of DOX inhibited TRIAP1 expression and induced cell death.^378^ Overexpressed circPVT1 increased the expression of TRIAP1 by competitively inhibiting miR‐137, inhibited caspase 3 cleavage maturation, and finally induced DOX‐resistance in SaOS cell lines (KHOS and U2OS).^376^ Epirubicin (EPI), an antibiotic antineoplastic drug, is an isomer of DOX that intercalates directly between DNA nucleobases and interferes with the transcription process.^417^ In SaOS, EPI can promote the expression of miR‐1301 and hinder the expression level of TRIAP1, thereby inhibiting the proliferation of U2OS and SAOS‐2 cell lines, promoting apoptosis, and exerting therapeutic effects.^377^

DDP, also known as cis‐dichlorodiammine platinum, is a platinum‐containing anticancer drug, which has a clinical curative effect on various solid tumors such as ovarian cancer, prostate cancer, and testicular cancer.^418^ In OC, the expression level of TRIAP1 was significantly higher in the DDP‐resistant SKOV3 cell line than in the DDP‐sensitive SKOV3 cell line.^44^ Highly expressed TRIAP1 induces DDP resistance from SKOV3 in vitro and in vivo by inhibiting mitochondrial cleavage and apoptosis.^44^ TKIs are effective targeted drugs for the treatment of various malignant tumors. TKIs can competitively bind ATP at the catalytic binding site of tyrosine kinases and inhibit tumor activity by inhibiting the phosphorylation process.^419^ In PCa, EGFR/RAS signaling upregulates TRIAP1 expression by inhibiting the miR‐203 expression and ultimately induces resistance to TKIs in a PCa cell line (RasB1).^382^ TAM is a selective estrogen receptor modulator that blocks estrogenic action in breast cells and activates estrogenic activity in other cells such as bone, liver, and uterine cells. Therefore, TAM may play a role in the treatment of cancer.^420^ Etoposide (VP‐16) is a commonly used antitumor chemotherapeutic drug that inhibits DNA replication, thereby inhibiting the cell cycle of cancer cells, and promoting cancer cell apoptosis and autophagy.^421^ In BrC, the expression level of TRIAP1 was significantly higher in TAM and VP‐16 resistant MCF7 cell lines than in sensitive MCF7 cell lines.^378^ PTX, a diterpene alkaloid compound with anticancer activity, is widely used in the treatment of breast, ovarian, and some head and neck, and lung cancers.^422^ In BrC, miR‐107 can inhibit TRIAP1 expression, sensitize the MCF‐7 cell line to PTX, and induce apoptosis.^379^

Radiation, consisting of the flow of atomic and subatomic particles and waves, including ionizing radiation, ultraviolet rays, and X‐rays, can be used in the treatment of various cancers.^423^ TRIAP1 enhances radiation resistance in NSCLC cell lines (A549 and H460),^366^ PCa cell lines (PC3), and CRC cell lines (HCT116).^41^ Radioresistant CAFs can promote radioresistance in PC3 xenograft tumors by secreting TRIAP1.^383^ At the same time, irradiation can upregulate the expression of TRIAP1 in NSCLC cell lines (A549 and H460), mediate the upregulation of various antioxidant proteins after irradiation, and finally induce ionizing radiation resistance.^366^

Therefore, therapeutic regimens targeting TRIAP1 may play an effective therapeutic role. We utilized Autodock Vina software (version 1.1.2, Linux) to perform batch molecular docking of TRIAP1 with 1438 FDA‐approved drugs to screen its targeted drugs. As shown in Table S3, 36 drugs were screened that could bind strongly to TRIAP1 (score < −7). As shown in Figure 6B, Ergotamine (score = −8.1) and Venetoclax (score = −8.5) could tightly bind to the surface of TRIAP1. This suggests that Ergotamine and Venetoclax are likely to be potential targeted inhibitors of TRIAP1.

### 6.5 Discussion

TRIAP1 is located at 12q24.31 and consists of two exons and one intron. TRIAP1 is a homolog of the yeast protein MDM35 with a similar structure and function. TRIAP1/PRELI heterodimers can transport phospholipids in the mitochondrial intermembrane space. TRIAP1 can exert an antiapoptotic effect by blocking mitochondria‐dependent apoptotic pathways.

TRIAP1 expression is strictly dependent on p53. The binding of p53 to the upstream sequence of TRIAP1 and the phosphorylation of p53 at different sites can have different effects on the expression of TRIAP1. In addition, the translation process of TRIAP1 was also inhibited by 12 miRNAs. Three ceRNAs can upregulate the expression level of TRIAP1 by competitively inhibiting miRNA. TRIAP1 was significantly positively correlated with the expression of most RNA‐modifying genes, indicating that TRIAP1 expression may be subjected to a large number of RNA modifications. But this still needs to be confirmed by experiments, which may be an important research direction for TRIAP1 expression regulation in the future.

TRIAP1 is highly expressed as an oncogene in most tumors and is only lowly expressed in PCPG. TRIAP1 plays an important role in the occurrence and development of cancer by inhibiting the apoptosis of cancer cells. Likewise, in most cancers, high expression of TRIAP1 is a high‐risk factor for patient prognosis. The expression level of TRIAP1 in cancer was significantly inversely correlated with ESTIMATE scores and positively correlated with CAFs activation status and tumor stemness. This may be the reason for the poor prognosis of patients with high expression of TRIAP1.

Interestingly, TRIAP1 was lowly expressed in PCPG and was hardly modified by RNA methylation. In PCPG, the TRIAP1 expression level was significantly positively correlated with ESTIMATE scores, and significantly negatively correlated with tumor stemness. Therefore, we speculate that TRIAP1 is subject to a unique regulatory mechanism in PCPG and thus plays different roles. Again, this still needs to be supported by experimental data. Elucidating the reason why TRIAP1 is different in PCPG from most other cancers may lead to a new understanding of TRIAP1 function.

Although there have been studies related to TRIAP1 and drug therapy, there is still no research and development of TRIAP1‐targeting drugs. As a tumor suppressor gene, TP53 can inhibit the proliferation of cancer cells and induce their apoptosis. However, TRIAP1 expression was significantly positively correlated with TP53 and mediated the antiapoptotic effect of TP53. Due to the particularity of the regulatory mechanism of TRIAP1 expression, high expression of TRIAP1 is likely to be the cause of drug resistance in most TP53‐targeted treatments. For example, sublethal doses of drugs may lead to phosphorylation of p53 Ser15, upregulate TRIAP1 expression, and induce cell resistance. Therefore, the drug treatment regimens around TRIAP1 and the treatment methods to avoid or reduce the development of drug resistance still need to be further studied. The development of TRIAP1‐targeted drugs is also imminent, which is also the focus and difficulty of future TRIAP1‐related research.

## 7 CONCLUSIONS AND PERSPECTIVE

p53 is encoded by the TP53 gene located at 17p13.1 and has 7 functional domains and 12 spliced isoforms. TP53 is the gene with the highest mutation frequency in tumors. The DBD missense mutation of TP53 will lead to abnormal function or inactivation of p53, which usually promotes tumorigenesis and development. The p53 pathway is a signaling pathway that receives and prevents information on cellular metabolic disorders and genetic material damage and can be activated when receiving stress signals. p53 is usually activated in the form of phosphorylation or acetylation, and inactivated in the form of ubiquitination or ubiquitin‐like. High levels of phosphorylated or acetylated p53 often predict better prognosis and survival in patients. Activated p53 forms a tetramer, binds to a variety of target genes, and promotes their transcription, thereby inhibiting cell carcinogenesis through various pathways, including blocking the cell cycle, inhibiting angiogenesis, regulating metabolism, promoting DNA damage repair, inducing cell apoptosis, and so on.

TRIAP1 is a novel p53‐dependent antiapoptotic gene that is highly expressed in various cancers. TRIAP1 is tightly regulated by p53 and mediates the antiapoptotic activity of p53. The expression of TRIAP1 is also subject to multiple epigenetic regulations, including RNA modification, and targeted binding of noncoding RNAs. Overexpression of TRIAP1 is a high‐risk factor affecting patient survival and is one of the reasons why many cancer cells develop drug resistance. With the progress of related treatment research and the development of targeted drugs, TRIAP1 is expected to play a vital role in clinical cancer treatment.

Although targeted therapy based on p53 signaling has been studied for decades, few drugs have been put into clinical trials or practical applications. Achieving targeted therapy of p53 signaling will be a disruptive achievement in cancer treatment research. There are various reasons why p53 signaling is currently difficult to target for therapy. For example, it may be due to differences in p53 mutation sites between different patients, or it may be the impact of differences in the expression of other genes on p53 signaling. There are still many gaps in the research on the p53 signaling pathway, such as the role of different mutations on p53 in different cancers, the phosphorylation or acetylation of different sites of p53 on the activation of different downstream factors, and so on. In the future, we need to know more about the impact of different site modifications on p53, in order to further explore the abnormal site of p53 signal in patients, so as to make a more precise treatment plan.

p53 can play a tumor suppressor role by activating multiple downstream genes, but some genes that are transcriptionally activated by p53 have tumor‐promoting activity, such as TRIAP1 and so on. TRIAP1 is a rare p53‐dependent oncogene that mediates the antiapoptotic activity of p53. Targeting TRIAP1, which is aberrantly expressed in cancer, is likely to be an effective cancer treatment option and has the potential to address resistance to p53 therapy. However, there are still many unknowns about the regulation of TRIAP1. In the future, relevant research needs to be carried out, such as the effect of phosphorylation at different sites of p53 on the expression of TRIAP1, the effect of other modifications of p53 on TRIAP1, whether TRIAP1 has feedback regulation on p53, and so on.

Therefore, ascertaining the p53 signaling pathway and continuously exploring new molecular mechanisms in p53 signaling is the research basis for targeted therapy. Individually differentiated treatment for p53 abnormalities in different patients will greatly improve the prognosis, survival time, and quality of life of cancer patients.

## AUTHOR CONTRIBUTION

J. S., Q. W., Y. M., W. G., and S. D. collected and analyzed the literature, drafted the figures, and wrote the paper; S. D. and W. G. conceived and gave the final approval of the submitted version. All authors have read and agreed to the published version of the manuscript.

## CONFLICT OF INTEREST STATEMENT

The authors declare that there are no competing interests.

## ETHICS STATEMENT

Not applicable.

## Supporting information

Supporting Information

## Data Availability

Not applicable.
