# Supplementary material for: Targeting the p53 signaling pathway in cancers: Molecular mechanisms and clinical studies
Source: MedComm (2020). 2023 May 28;4(3):e288. doi: 10.1002/mco2.288 (PMC10225743; doi:10.1002/mco2.288)
Supplement: Supplementary file 1 — Supporting Information [file MCO2-4-e288-s001.pdf]

Targeting the p53 signaling pathway in cancers: molecular mechanisms and clinical studies

Jinze Shen[1], Qurui Wang[1], Yunan Mao[1], Wei Gao[1,*], Shiwei Duan[1,*]

1. Key Laboratory of Novel Targets and Drug Study for Neural Repair of Zhejiang Province, School of Medicine, Hangzhou City University, Hangzhou, Zhejiang, 310015, China

*: Correspondence should be addressed to Drs. Shiwei Duan (duansw@zucc.edu.cn) and Wei Gao (gaow@zucc.edu.cn).

Table of contents

Text

1. A pan-cancer analysis of TRIAP1 based on TCGA database (Page 2)

2. TRIAP1 expression may be regulated by RNA modifications (Pages 2-3)

3. Prognostic study of TRIAP1 based on TCGA database (Page 3)

4. TRIAP1 and the tumor microenvironment (TME) (Pages 3-4)

5. TRIAP1 and tumor stemness (Page 4)

Tables

Table S1. The binding sites of TRIAP1 and miRNA (Page 5)

Table S2. TRIAP1 up-regulated in drug-resistant cell lines (Page 6)

Table S3. Predicted drugs capable of strong binding to TRIAP1 (Pages 7-8)

References (Pages 9-10)

Figures

Figure S1. TRIAP1 expression is dependent on p53 (Page 11)

Figure S2. The correlation of TRIAP1 expression and RNA modifying genes (Page 12)

Figure S3. The contribution of TRIAP1 to cancer prognosis (Page 13)

Text

1. A pan-cancer analysis of TRIAP1 based on TCGA database

TRIAP1 is an anti-apoptotic factor, and experiments have shown that the expression level of TRIAP1 is generally elevated in 9 types of cancers. To further explore the expression changes of TRIAP1 in more types of cancers, we downloaded the expression data (TPM) of TRIAP1 in the TCGA (Pan-cancer) and GTEx datasets (https://xenabrowser.net/) from the UCSC Xena database. We removed the samples with zero expressions and performed a log2 (TPM+0.001) transformation of the expression level to obtain the expression data of TRIAP1 in 33 tumors. We divided all samples into the tumor group (from TCGA) and normal group (from TCGA and GTEx), and performed between-group tests using an unpaired Wilcoxon test. As shown in Figure 3, TRIAP1 was significantly up-regulated in 26 tumors (Bonferroni-adjusted $p < 0.05$), significantly down-regulated only in PCPG (Bonferroni-adjusted $p < 0.05$), and not significantly different in 3 tumors (KICH, READ, and THYM).

To further explore the correlation between TRIAP1 and TP53 expression levels in pan-cancer, we downloaded the TP53 expression data (TPM) in TCGA (Pan-cancer) dataset from the UCSC Xena database and performed log2(TPM+0.001) transformation. We calculated the correlation between the expression levels of TP53 and TRIAP1 in each tumor in situ tissue by Pearson's test. Our results showed that TRIAP1 expression levels were significantly positively correlated with TP53 in 15 tumors (Figure S1, Bonferroni adjusted $p < 0.05$ and $r > 0.25$).

To further explore the effect of TP53 gene mutation on TRIAP1 expression, we downloaded the TP53 mutation data in the TCGA (Pan-cancer) dataset from the UCSC Xena database. We found that in 3 tumors (COAD, HNSC, and LGG), TP53 mutation resulted in a significant downregulation of TRIAP1 expression (Figure S1, Wilcoxon-Test, adjusted $p < 0.05$). This may be due to p53 mutation leading to decreased p53 function, which in turn reduces the level of TRIAP1 expression.

In conclusion, our bioinformatics analysis verified that TRIAP1 expression was significantly dependent on p53 protein.

2. TRIAP1 expression may be regulated by RNA modifications

RNA modification refers to various covalent modifications that occur on RNA, which have the effect of changing RNA stability and regulating RNA translation [1]. Among them, methylation

modification is the most extensive form of RNA modification, including N(6)-methyladenosine (m6A), 5-methylcytosine (m5C), and N1-methyladenosine (m1A) [2]. We analyzed the correlation of RNA-modifying genes with TRIAP1 expression levels. The results showed that TRIAP1 was significantly associated with most RNA-modifying genes in most cancers (Figure S2, Bonferroni-adjusted $p < 0.05$ and $|r| > 0.3$), suggesting that the expression of TRIAP1 in these cancers may be regulated by RNA modification.

3. Prognostic study of TRIAP1 based on TCGA database

We downloaded patient survival (OS and DSS) data in TCGA (Pan-cancer) from the UCSC Xena database. We calculated the risk score of TRIAP1 in pan-cancer using univariate COX analysis. As shown in Figure S3a, among the seven tumors, patients with high TRIAP1 expression had a higher hazard ratio (HR) ($p < 0.05$) for overall survival (OS). In addition, disease-specific survival (DSS) of patients with high TRIAP1 expression in 8 tumors had higher HR ($p < 0.05$). These findings support previous results suggesting that TRIAP1 can serve as a high-risk marker for diagnosis and prognosis in these cancers.

4. TRIAP1 and the tumor microenvironment (TME)

TME refers to the surrounding microenvironment in which tumor cells exist, including peripheral blood vessels, immune cells, stromal cells, and extracellular matrix. TME has a regulatory effect on the occurrence and development of tumors [3].

Infiltrating stromal cells and immune cells are important components of the TME and have important roles in regulating immune responses and interfering with tumor signaling [4]. ESTIMATE is a method for calculating the degree of stromal and immune cell infiltration using gene expression signatures [5]. We calculated the correlation between the expression level of TRIAP1 and the stromal, immune, and ESTIMATE scores of each sample in each tumor in situ tissue. The results are shown in Figure S3b. In 3 tumors (ESCA, LUSC, and TGCT), TRIAP1 expression levels were significantly inversely correlated with stromal, immune, and ESTIMATE scores. In UCEC, TRIAP1 expression levels were significantly inversely correlated with stromal and ESTIMATE scores. In 4 tumors (CESC, CHOL, LUAD, and THYM), TRIAP1 expression levels were significantly inversely correlated with immune and ESTIMATE scores ($p < 0.05$ and $|r| > 0.2$).

Cancer-associated fibroblasts (CAFs) are the most abundant stromal cells in the TME and are the communication center of various cells in the TME [6]. CAFs can secrete a variety of active factors, promote tumor progression, and are closely related to treatment resistance [7]. We downloaded the expression data (TPM) of CAF hallmarks (ACTA2, FAP, PDGFRA, PDGFRB, PDPN, THY1, and COL1A1) [8] from the UCSC Xena database (https://xenabrowser.net/), and log2(TPM+0.001) transformation was performed on the gene expression. We calculated the correlation between the expression levels of TRIAP1 and CAF hallmarks in each tumor. As shown in Figure S3b, TRIAP1 was significantly positively correlated with more than 2 CAF hallmarks in 10 tumors ($p < 0.05$ and $|r| > 0.25$).

In TCGA, TRIAP1 was inversely correlated with ESTIMATE score and positively correlated with CAF hallmarks. Low activity of antitumor immune cells and high activity of CAFs may lead to high expression of TRIAP1 in tumors, which in turn leads to poor prognosis of patients. In PCPG, the expression level of TRIAP1 was positively correlated with both stromal and ESTIMATE scores ($p < 0.05$ and $|r| > 0.25$). This is consistent with the low expression of TRIAP1 in PCPG mentioned above, suggesting that TRIAP1 is subject to an unknown and unique regulatory mechanism, thus playing different roles in PCPG.

5. TRIAP1 and tumor stemness

Tumor stemness is an indicator closely related to cell proliferation, which can predict the ability of tumor cell self-renewal and heterogeneity of tumor cells [9]. Tumor stemness plays an important role in malignant tumor progression, metastasis, and recurrence, and is an important factor in the poor prognosis of tumor patients [10]. We obtained tumor stemness score data (including DNAss and RNAss) from the UCSC Xena database (https://xenabrowser.net/) [11]. We calculated the correlation of TRIAP1 expression with DNA methylation-based stemness score (DNAss) and RNA expression-based stemness score (RNAss) in each tumor. The results are shown in Figure S3c. In 4 tumors (ESCA, HNSC, LUSC, and LGG), TRIAP1 was significantly positively correlated with DNAss. In 15 tumors, there was a significant positive correlation with RNAss ($p < 0.05$ and $r > 0.2$), which may also be one of the reasons for the poor prognosis of patients with high TRIAP1 expression. Likewise, TRIAP1 was significantly inversely correlated with DNAss and RNAss in PCPG ($p < 0.05$ and $|r| > 0.2$).

Tables

Table S1. The binding sites of TRIAP1 and miRNA

miRNA	Disease	Binding site of miRNA and TRIAP1		Ref.
		PCG (5'-…-3')	miRNA (3'-…-5')	
miR-18a	EOC	UGCuuUuGAGCACaCUU	ACGugAuCUaCGUGGAA	[12]
miR-107	BrC	AAUGCUGC	UUACGACG	[13]
	GC	—	—	[14]
	NSCLC	AUGCUGC	UACGACG	[15]
	OSCC	UUACGACGA	AAUGCUGCA	[16]
miR-125a-5p	HCC	CUCAGGG	GAGUCCC	[17]
	TC	UCUCAGGGA	AGAGUCCCU	[18]
miR-125b-5p	LDD	CUCAGGG	GAGUCCC	[19]
miR-137	SaOS	GCAAUA	CGUUAU	[20]
miR-203	PCa	GUAAAGU	CAUUUCA	[21]
miR-320b	NPC	CAGCUUU	GUCGAAA	[22]
miR-320d	AD	—	—	[23]
miR-506	PCa	AAGGCA	UUCCGU	[24]
miR-539	SaOS	AUUUCUC	UAAAGAG	[25]
miR-770-5p	DN	ACACuGUACUGG	UGUGcacCAUGACC	[26]
	GDM	ACACuGUACUGG	UGUGcacCAUGACC	[27]
miR-1301	SaOS	AGCUGCAA	UCGACGUU	[28]

EOC, epithelial ovarian cancer; GC, gastric cancer; BrC, breast cancer; NSCLC, non-small-cell lung cancer; OSCC, oral squamous cell carcinoma; TC, thyroid cancer; HCC, hepatocellular carcinoma; LDD, lumbar disc degeneration; SaOS, osteosarcoma; PCa, prostate cancer; NPC, nasopharyngeal carcinoma; AD, aortic dissection; DN, diabetic nephropathy; GDM, gestational diabetes mellitus.

Table S2. TRIAP1 is up-regulated in drug-resistant cell lines

Disease	Expression in resistant cell line	Sensitive cell line	Resistant cell line	Ref.
SaOS	Up-regulated	KHOS	KHOS/DOX	[20]
	Up-regulated	U2OS	U2OS/DOX	
OC	Up-regulated	SKOV3	SKOV3/DDP	[29]
BrC	Up-regulated	CAL51	CAL51/DOX	[30]
	Up-regulated	MCF7	MCF7/DOX	
			MCF7/TAM and VP-16	

DOX, doxorubicin; DDP, cisplatin; TAM, tamoxifen; VP-16, etoposide.

Table S3. Predicted drugs that bind strongly to TRIAP1

ZINC ID	international non-proprietary name	PubChem CID	CAS number	AutoDock Vina score (kcal/mol)
ZINC000150338755	Venetoclax	49846579	1257044-40-8	-8.5
ZINC000052955754	Ergotamine	8223	113-15-5	-8.1
ZINC000150338819	Ledipasvir	67505836	1256388-51-8	-8.1
ZINC000164760756	Olysio	24873435	923604-59-5	-8.1
ZINC000012503187	Conivaptan	151171	210101-16-9	-8
ZINC000003978005	Dihydroergotamine	10531	511-12-6	-7.9
ZINC000003927200	Yaz	147740	164017-31-6	-7.8
ZINC000036701290	Ponatinib	24826799	943319-70-8	-7.8
ZINC000006716957	Nilotinib	644241	641571-10-0	-7.6
ZINC000064033452	Lumacaftor	16678941	936727-05-8	-7.6
ZINC000100013130	Midostaurin	9829523	120685-11-2	-7.6
ZINC000003932831	Avodart	6918296	164656-23-9	-7.5
ZINC000027990463	Lomitapide	9853053	182431-12-5	-7.5
ZINC000001612996	Irinotecan	60838	97682-44-5	-7.4
ZINC000003985982	Inspra	443872	107724-20-9	-7.4
ZINC000006745272	Stivarga	1116760	755037-03-7	-7.4
ZINC000003816514	Rolapitant	10311306	552292-08-7	-7.3
ZINC000003927822	Lurasidone	213046	367514-87-2	-7.3
ZINC000011679756	Eltrombopag	135449332	496775-61-2	-7.3
ZINC000150588351	Elbasvir	71661251	1370468-36-2	-7.3
ZINC000169289767	Trypan Blue	6296	72-57-1	-7.3
ZINC000022448696	Indinavir	5362440	150378-17-9	-7.2
ZINC000003817234	Celsentri	3002977	376348-65-1	-7.1
ZINC000003993846	Ixabepilone	6445540	219989-84-1	-7.1
ZINC000052245489	Ixempra	6445540	219989-84-1	-7.1
ZINC000003820029	Trajenta	10096344	668270-12-0	-7
ZINC000003915154	Alvesco	6918155	126544-47-6	-7
ZINC000003938482	Noxafil	468595	171228-49-2	-7
ZINC000011681563	Netupitant	6451149	290297-26-6	-7
ZINC000014261579	Alvesco	6918155	126544-47-6	-7
ZINC000019632618	Imatinib	5291	152459-95-5	-7
ZINC000028639340	Noxafil	468595	171228-49-2	-7
ZINC000068204830	Daclatasvir	25154714	1009119-64-5	-7
ZINC000084668739	Lifitegrast	11965427	1025967-78-5	-7
ZINC000100370145	Ecamsule	71587347	92841-53-7	-7
ZINC000100378061	Naldemedine	54732242	916072-89-4	-7

We obtained the predicted protein structure of TRIAP1 from the AlphaFold Protein Structure Database (https://alphafold.ebi.ac.uk/) and subjected the protein to combined charges, removal of lone pair electrons and water, and addition of polar hydrogens. Meanwhile, we obtained the 3D structures of 1438 FDA-approved drugs from the ZINC15 database (https://zinc.docking.org/) and performed batch

molecular docking for drug screening using Autodock Vina software (version 1.1.2, Linux). Thirty-six drugs (score < -7) that could bind strongly to TRIAP1 were screened.

References

1. Shi B, Liu WW, Yang K, Jiang GM, Wang H. The role, mechanism, and application of RNA methyltransferase METTL14 in gastrointestinal cancer. *Mol Cancer*. 2022;21(1):163.
2. Zhang T, Gu J, Wang X, et al. RNA methylation regulators contribute to poor prognosis of hepatocellular carcinoma associated with the suppression of bile acid metabolism: a multi-omics analysis. *Am J Cancer Res*. 2022;12(7):2989-3013.
3. Ullman NA, Burchard PR, Dunne RF, Linehan DC. Immunologic Strategies in Pancreatic Cancer: Making Cold Tumors Hot. *J Clin Oncol*. 2022;40(24):2789-2805.
4. Plava J, Cihova M, Burikova M, Matuskova M, Kucerova L, Miklikova S. Recent advances in understanding tumor stroma-mediated chemoresistance in breast cancer. *Mol Cancer*. 2019;18(1):67.
5. Yoshihara K, Shahmoradgoli M, Martinez E, et al. Inferring tumour purity and stromal and immune cell admixture from expression data. *Nat Commun*. 2013;4:2612.
6. Chen X, Song E. Turning foes to friends: targeting cancer-associated fibroblasts. *Nat Rev Drug Discov*. 2019;18(2):99-115.
7. Saw PE, Chen J, Song E. Targeting CAFs to overcome anticancer therapeutic resistance. *Trends Cancer*. 2022;8(7):527-555.
8. Zheng S, Zou Y, Tang Y, et al. Landscape of cancer-associated fibroblasts identifies the secreted biglycan as a protumor and immunosuppressive factor in triple-negative breast cancer. *Oncoimmunology*. 2022;11(1):2020984.
9. Xuan W, Khan F, James CD, Heimberger AB, Lesniak MS, Chen P. Circadian regulation of cancer cell and tumor microenvironment crosstalk. *Trends Cell Biol*. 2021;31(11):940-950.
10. Yamashita T, Wang XW. Cancer stem cells in the development of liver cancer. *J Clin Invest*. 2013;123(5):1911-8.
11. Malta TM, Sokolov A, Gentles AJ, et al. Machine Learning Identifies Stemness Features Associated with Oncogenic Dedifferentiation. *Cell*. 2018;173(2):338-354 e15.
12. Liu P, Qi X, Bian C, et al. MicroRNA-18a inhibits ovarian cancer growth via directly targeting TRIAP1 and IPMK. *Oncol Lett*. 2017;13(6):4039-4046.
13. Luo Y, Hua T, You X, Lou J, Yang X, Tang N. Effects of MiR-107 on The Chemo-drug Sensitivity of Breast Cancer Cells. *Open Med (Wars)*. 2019;14:59-65.
14. Yan J, Dai L, Yuan J, et al. miR-107 Inhibits the Proliferation of Gastric Cancer Cells In vivo and In vitro by Targeting TRIAP1. *Front Genet*. 2022;13:855355.
15. Cai P, Li J, Chen G, et al. MicroRNA-107 may regulate lung cancer cell proliferation and apoptosis by targeting TP53 regulated inhibitor of apoptosis 1. *Oncol Lett*. 2020;19(3):1958-1966.
16. Na C, Li X, Zhang J, Han L, Li Y, Zhang H. miR-107 targets TRIAP1 to regulate oral squamous cell carcinoma proliferation and migration. *Int J Clin Exp Pathol*. 2019;12(5):1820-1825.
17. Ming M, Ying M, Ling M. miRNA-125a-5p inhibits hepatocellular carcinoma cell proliferation and induces apoptosis by targeting TP53 regulated inhibitor of apoptosis 1 and Bcl-2-like-2 protein. *Exp Ther Med*. 2019;18(2):1196-1202.
18. Yu T, Tong L, Ao Y, Zhang G, Liu Y, Zhang H. Upregulation of TRIAP1 by the lncRNA MFI2-AS1/miR-125a-5p Axis Promotes Thyroid Cancer Tumorigenesis. *Onco Targets Ther*. 2020;13:6967-6974.
19. Jie J, Xu X, Li W, Wang G. Regulation of Apoptosis and Inflammatory Response in Interleukin-1beta-Induced Nucleus Pulposus Cells by miR-125b-5p Via Targeting TRIAP1. *Biochem Genet*.

2021;59(2):475-490.
20. Li D, Huang Y, Wang G. Circular RNA circPVT1 Contributes to Doxorubicin (DXR) Resistance of Osteosarcoma Cells by Regulating TRIAP1 via miR-137. *Biomed Res Int*. 2021;2021:7463867.
21. Siu MK, Abou-Kheir W, Yin JJ, et al. Loss of EGFR signaling regulated miR-203 promotes prostate cancer bone metastasis and tyrosine kinase inhibitors resistance. *Oncotarget*. 2014;5(11):3770-84.
22. Li Y, Tang X, He Q, et al. Overexpression of Mitochondria Mediator Gene TRIAP1 by miR-320b Loss Is Associated with Progression in Nasopharyngeal Carcinoma. *PLoS Genet*. 2016;12(7):e1006183.
23. Shen H, Lu S, Dong L, et al. hsa-miR-320d and hsa-miR-582, miRNA Biomarkers of Aortic Dissection, Regulate Apoptosis of Vascular Smooth Muscle Cells. *J Cardiovasc Pharmacol*. 2018;71(5):275-282.
24. Liu H, He X, Li T, et al. PCGEM1 promotes proliferation, migration and invasion in prostate cancer by sponging miR-506 to upregulate TRIAP1. *BMC Urol*. 2022;22(1):14.
25. Liu H, Yang M, Zhang Y, et al. The effect of miR-539 regulating TRIAP1 on the apoptosis, proliferation, migration and invasion of osteosarcoma cells. *Cancer Cell Int*. 2021;21(1):227.
26. Zhang SZ, Qiu XJ, Dong SS, et al. MicroRNA-770-5p is involved in the development of diabetic nephropathy through regulating podocyte apoptosis by targeting TP53 regulated inhibitor of apoptosis 1. *Eur Rev Med Pharmacol Sci*. 2019;23(3):1248-1256.
27. Zhang YL, Chen XQ. Dysregulation of microRNA-770-5p influences pancreatic-beta-cell function by targeting TP53 regulated inhibitor of apoptosis 1 in gestational diabetes mellitus. *Eur Rev Med Pharmacol Sci*. 2020;24(2):793-801.
28. Yu L, Meng M, Bao Y, et al. miR-1301/TRIAP1 Axis Participates in Epirubicin-Mediated Anti-Proliferation and Pro-Apoptosis in Osteosarcoma. *Yonsei Med J*. 2019;60(9):832-841.
29. Zhang TM. TRIAP1 Inhibition Activates the Cytochrome c/Apaf-1/Caspase-9 Signaling Pathway to Enhance Human Ovarian Cancer Sensitivity to Cisplatin. *Chemotherapy*. 2019;64(3):119-128.
30. Adams C, Cazzanelli G, Rasul S, et al. Apoptosis inhibitor TRIAP1 is a novel effector of drug resistance. *Oncol Rep*. 2015;34(1):415-22.

Figures

Figure S1. TRIAP1 expression is dependent on p53

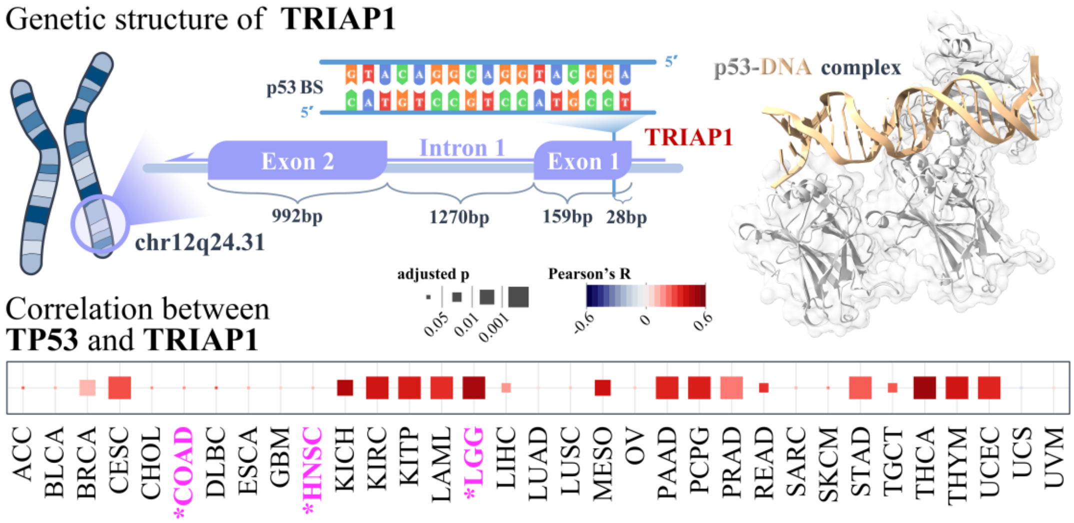

TRIAP1 is located in the minus strand of 12q24.31 and consists of 2 exons and 1 intron. A potential p53-binding sequence (p53 BS) is present in intron 1. In 15 tumors (CESC, KICH, KIRC, KIRP, LAML, LGG, MESO, PAAD, PCPG, READ, STAD, TGCT, THCA, THYM, and UCEC), TRIAP1 expression levels were significantly positively correlated with TP53 (Pearson test, adjusted $p < 0.05$ and $r > 0.25$). Tumors marked with an asterisk (magenta) indicate significantly decreased TRIAP1 expression in the TP53 mutant group in this tumor. In 3 tumors (COAD, HNSC, and LGG), TRIAP1 expression was significantly decreased in the TP53 mutant group (Wilcoxon-Test, adjusted $p < 0.05$). Please check GDC (https://gdc.cancer.gov/resources-tcga-users/tcga-code-tables/tcga-study-abbreviations) for the full name of the TCGA abbreviations.

Figure S2. The correlation of TRIAP1 and RNA-modifying genes

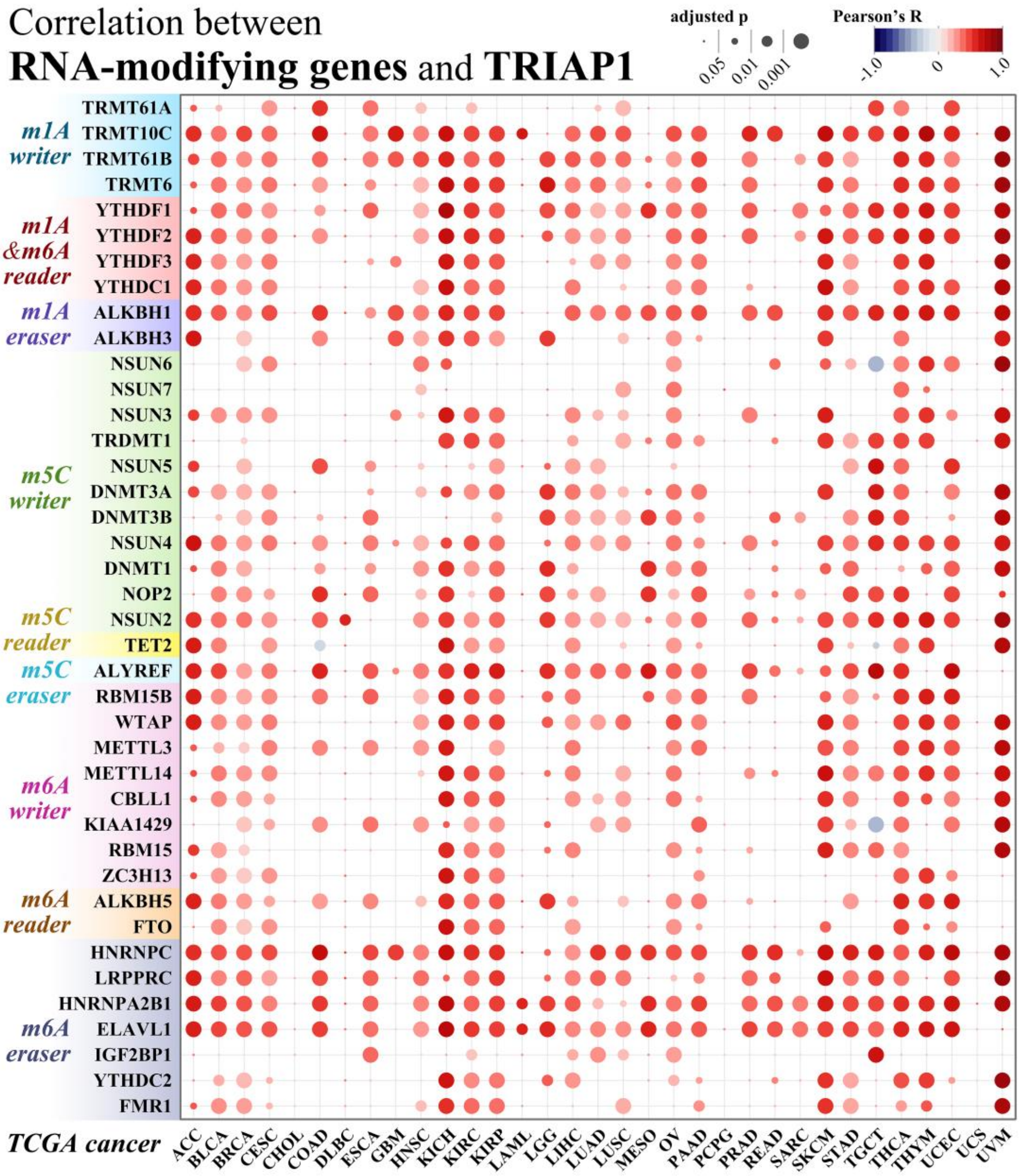

We downloaded the expression data (TPM) of 40 RNA-modifying genes in TCGA (Pan-cancer) from the UCSC Xena database and performed log2(TPM+0.001) transformation. We analyzed the correlation of RNA-modifying genes with TRIAP1. TRIAP1 was significantly associated with most RNA-modifying genes, indicating that the expression of TRIAP1 in these cancers may be regulated by a large number of RNA modifications. Only in CHOL, DLBC, PCPG, and UCS, TRIAP1 was not significantly associated with RNA-modifying genes (adjusted $p > 0.05$ and $|r| < 0.3$). Please check GDC (https://gdc.cancer.gov/resources-tcga-users/tcga-code-tables/tcga-study-abbreviations) for the full name of the TCGA abbreviations.

Figure S3. The contribution of TRIAP1 to cancer prognosis

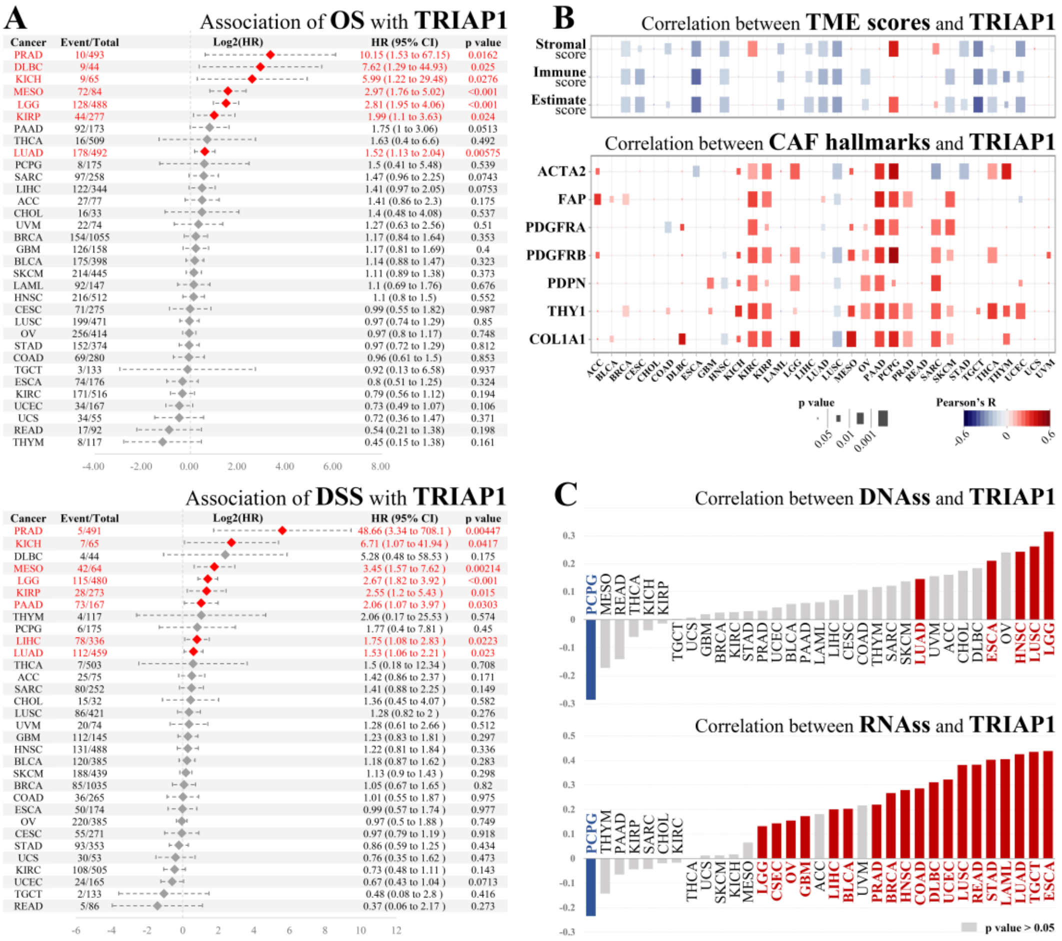

(A) In a variety of cancers, high expression of TRIAP1 can be an independent risk factor for patient prognosis. In TCGA data, high expression of TRIAP1 is a high-risk factor for cancer patient survival. High expression of TRIAP1 results in poorer OS in 7 tumors (PRAD, DLBC, KICH, MESO, LGG, KIRP, and LUAD) and poorer DSS in 8 tumors (PRAD, KICH, MESO, LGG, KIRP, PAAD, LIHC, and LUAD) (HR > 1 and $p < 0.05$). This is consistent with the results mentioned in the main text that the expression of TRIAP1 in these cancers is higher than the corresponding normal group (except DLBC and MESO without the control group).

(B) TRIAP1 may mediate the effect of immune microenvironmental changes on tumor progression.

(C) TRIAP1 is significantly positively correlated with tumor stemness (including DNAss and RNAss) in various cancers, which may be one of the reasons for the poor prognosis of patients with high TRIAP1 expression. TRIAP1 was inversely correlated with tumor stemness score only in PCPG, which was consistent with the down-regulation of TRIAP1 in PCPG, indicating that TRIAP1 may have a special molecular mechanism of action in PCPG.

OS, overall survival; DSS, disease-specifc survival; HR, hazard ratio; TME, tumor micro-environment; CAF, cancer-associated fibroblast; DNAss, DNA methylation-based stemness score; RNAss, RNA expression-based stemness score. Please check GDC (https://gdc.cancer.gov/resources-tcga-users/tcga-code-tables/tcga-study-abbreviations) for the full name of the TCGA abbreviations.
